# Fonio millet genome unlocks African orphan crop diversity for agriculture in a changing climate

**DOI:** 10.1038/s41467-020-18329-4

**Published:** 2020-09-08

**Authors:** Michael Abrouk, Hanin Ibrahim Ahmed, Philippe Cubry, Denisa Šimoníková, Stéphane Cauet, Yveline Pailles, Jan Bettgenhaeuser, Liubov Gapa, Nora Scarcelli, Marie Couderc, Leila Zekraoui, Nagarajan Kathiresan, Jana Čížková, Eva Hřibová, Jaroslav Doležel, Sandrine Arribat, Hélène Bergès, Jan J. Wieringa, Mathieu Gueye, Ndjido A. Kane, Christian Leclerc, Sandrine Causse, Sylvie Vancoppenolle, Claire Billot, Thomas Wicker, Yves Vigouroux, Adeline Barnaud, Simon G. Krattinger

**Affiliations:** 1 Center for Desert Agriculture, Biological and Environmental Science & Engineering Division (BESE), King Abdullah University of Science and Technology (KAUST), Thuwal, Saudi Arabia; 2 DIADE, Univ Montpellier, IRD, Montpellier, France; 3 Institute of Experimental Botany of the Czech Academy of Sciences, Centre of the Region Hana for Biotechnological and Agricultural Research, Olomouc, Czech Republic; 4 CNRGV Plant Genomics Center, INRAE, Toulouse, France; 5 Supercomputing Core Lab, King Abdullah University of Science and Technology (KAUST), Thuwal, Saudi Arabia; 6 Inari Agriculture, One Kendall Square Building 600/700, Cambridge, MA 02139 USA; 7 Naturalis Biodiversity Center, Leiden, the Netherlands; 8 Laboratoire de Botanique, Département de Botanique et Géologie, IFAN Ch. A. Diop/UCAD, Dakar, Senegal; 9 Senegalese Agricultural Research Institute, Dakar, Senegal; 10 Laboratoire Mixte International LAPSE, Dakar, Senegal; 11 CIRAD, UMR AGAP, Montpellier, France; 12 AGAP, Université de Montpellier, Cirad, INRAE, Institut Agro, Montpellier, France; 13 Department of Plant and Microbial Biology, University of Zurich, Zürich, Switzerland

**Keywords:** Agricultural genetics, Genetic variation, Plant breeding, Plant domestication

## Abstract

Sustainable food production in the context of climate change necessitates diversification of agriculture and a more efficient utilization of plant genetic resources. Fonio millet (*Digitaria exilis*) is an orphan African cereal crop with a great potential for dryland agriculture. Here, we establish high-quality genomic resources to facilitate fonio improvement through molecular breeding. These include a chromosome-scale reference assembly and deep re-sequencing of 183 cultivated and wild *Digitaria* accessions, enabling insights into genetic diversity, population structure, and domestication. Fonio diversity is shaped by climatic, geographic, and ethnolinguistic factors. Two genes associated with seed size and shattering showed signatures of selection. Most known domestication genes from other cereal models however have not experienced strong selection in fonio, providing direct targets to rapidly improve this crop for agriculture in hot and dry environments.

## Abstract (web summary)

Fonio millet is a fast growing orphan cereal crop with a great potential for dryland agriculture. Here, the authors report chromosome-scale reference genome assembly and population genomic resources to shed light on genetic diversity, population structure and domestication of fonio millet.

## Introduction

Humanity faces the unprecedented challenge of having to sustainably produce healthy food for 9–10 billion people by 2050 in a context of climate change. A more efficient use of plant diversity and genetic resources in breeding has been recognized as a key priority to diversify and transform agriculture^1–3^. The Food and Agriculture Organization of the United Nations (FAO) stated that arid and semi-arid regions are the most vulnerable environments to increasing uncertainties in regional and global food production^4^. In most countries of Africa and the Middle East, agricultural productivity will decline in the near future^4^, because of climate change, land degradation, and groundwater depletion^5^. Agricultural selection, from the early steps of domestication to modern-day crop breeding, has resulted in a marked decrease in agrobiodiversity^6,7^. Today, three cereal crops alone, bread wheat (*Triticum aestivum*), maize (*Zea mays*), and rice (*Oryza sativa*) account for more than half of the globally consumed calories^8^.

Many of today’s major cereal crops, including rice and maize, originated in relatively humid tropical and sub-tropical regions^9,10^. Although plant breeding has adapted the major cereal crops to a wide range of climates and cultivation practices, there is limited genetic diversity within these few plant species for cultivation in the most extreme environments. On the other hand, crop wild relatives and orphan crops are often adapted to extreme environments and their utility to unlock marginal lands for agriculture has recently regained interest^2,6,11–14^. Current technological advances in genomics and genome editing provide an opportunity to rapidly domesticate wild relatives and to improve orphan crops^15,16^. De novo domestication of wild species or rapid improvement of semi-domesticated crops can be achieved in less than a decade by targeting a few key genes^6^.

**Fig. 1:**
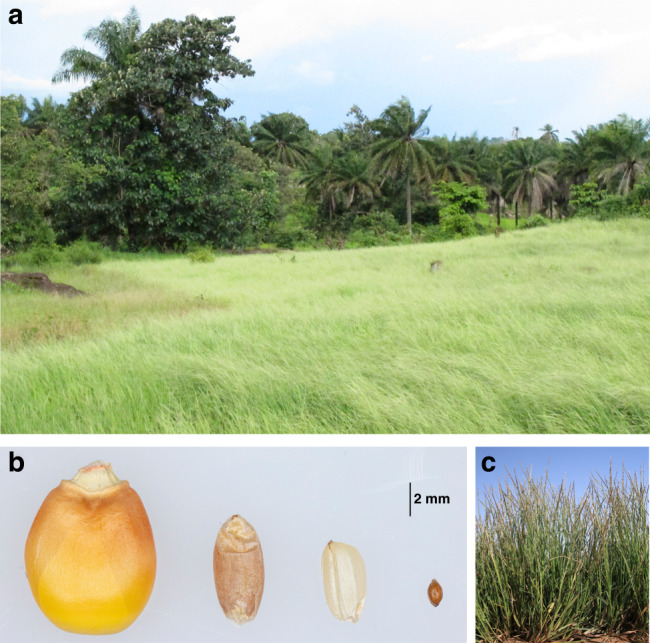
Phenotype of fonio (*Digitaria exilis*). **a** Field of cultivated fonio in Guinea. **b** Grains of maize, wheat, rice, and fonio (from left to right). **c** Plants of the fonio accession CM05836.

White fonio (*Digitaria exilis* (Kippist) Stapf) (Fig. 1) is an indigenous West African millet species with a great potential for agriculture in marginal environments^17,18^. Fonio is a small annual herbaceous C4 plant, which produces very small (∼1 mm) grains that are tightly surrounded by a husk^19^ (Fig. 1). Fonio is cultivated under a large range of environmental conditions, from a tropical monsoon climate in western Guinea to a hot, arid desert climate in the Sahel zone. Some extra-early maturing fonio varieties produce mature grains in only 70–90 days^19^, which makes fonio one of the fastest maturing cereals. Because of its quick maturation, fonio is often grown to avoid food shortage during the lean season (period before main harvest), which is why fonio is also referred to as ‘hungry rice’. In addition, fonio is drought tolerant and adapted to nutrient-poor, sandy soils^20^. Despite its local importance for agriculture in West Africa, fonio shows many unfavorable characteristics that resemble undomesticated plants: residual seed shattering, lodging, and lower yields than other cereals^18^ (Supplementary Fig. 1). The most likely wild progenitor of cultivated fonio is the tetraploid annual weed *D. longiflora* that is widely distributed in tropical Africa. It has been suggested that fonio was domesticated more than 5000 years ago in the Inner Niger Delta of central Mali. Compared to *D. exilis*, the wild *D. longiflora* shows even heavier seed shattering and hairy spikelets, which suggests that some selection for reduced seed shattering and spikelet hairiness occurred in fonio^21^. In the past few years, fonio has gained in popularity inside and outside of West Africa because of its nutritional qualities.

Here, we present the establishment of a comprehensive set of genomic resources for fonio, which constitutes the first step towards harnessing the potential of this cereal crop for agriculture in harsh environments. These resources include the generation of a high-quality, chromosome-scale reference assembly and the deep re-sequencing of a diversity panel that includes wild and cultivated accessions covering a wide geographic range.

## Results

### Chromosome-scale fonio reference genome assembly

**Fig. 2:**
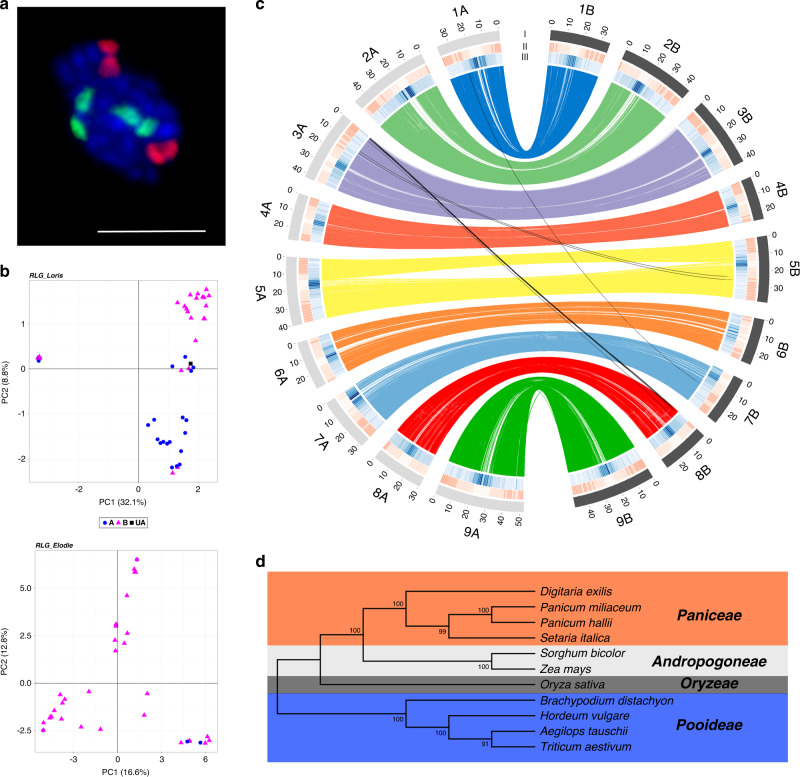
Fonio genome features. **a** Representative example of oligo painting FISH on mitotic metaphase chromosomes. Shown are probes designed from pseudomolecules 9A (green) and 9B (red) of the CM05836 assembly (scale bar = 5 µm). Oligo painting FISH experiment was repeated independently three times. **b** Principal component analysis (PCA) of the transposable element cluster RLG_Loris (upper panel) that allowed discrimination of the two sub-genomes. Blue dots represent elements found on the A sub-genome; pink triangles represent elements from the B sub-genome; black squares represent elements present on chromosome unanchored. PCA of the transposable element cluster RLG_Elodie (lower panel) that was specific to the B sub-genome. **c** Synteny and distribution of genome features. (I) Number and length of the pseudomolecules. The gray and black colors represent the two sub-genomes. (II, III) Density of genes and repeats along each pseudomolecule, respectively. Lines in the inner circle represent the homoeologous relationships. **d** Maximum likehood tree of 11 *Poaceae* species based on 30 orthologous gene groups. Topologies are supported by 1000 bootstrap replicates. Colors indicate the different clades.

Fonio is a tetraploid species (2*n* = 4× = 36)^22^ with a highly inbreeding reproductive system^17^. To build a *D. exilis* reference assembly, we chose an accession from one of the driest regions of fonio cultivation, CM05836 from the Mopti region in Mali. The size of the CM05836 genome was estimated to be 893 Mb/1C by flow cytometry (Supplementary Figs. 2 and 3), which is in line with previous reports^22^. The CM05836 genome was sequenced and assembled using deep sequencing of multiple short-read libraries (Supplementary Table 1), including Illumina paired-end (321-fold coverage), mate-pair (241-fold coverage) and linked-read (10× Genomics, 84-fold coverage) sequencing. The raw reads were assembled and scaffolded with the software package DeNovoMAGIC3 (NRGene), which has recently been used to assemble various high-quality plant genomes^23–25^. Integration of Hi-C reads (122-fold coverage, Supplementary Table 2) and a Bionano optical map (Supplementary Table 3) resulted in a chromosome-scale assembly with a total length of 716,471,022 bp, of which ~91.5% (655,723,161 bp) were assembled in 18 pseudomolecules. A total of 60.75 Mb were unanchored (Table 1). Of 1440 Embryophyta single copy core genes (BUSCO v3.0.2), 96.1% were recovered in the CM05836 assembly, 2.9% were missing, and 1% was fragmented. As no genetic *D. exilis* map is available, we used chromosome painting to further assess the quality of the CM05836 assembly. Pools of short oligonucleotides covering each one of the 18 pseudomolecules were designed based on the CM05836 assembly, fluorescently labeled, and hybridized to mitotic metaphase chromosome spreads of CM05836^26^. Each of the 18 libraries specifically hybridized to only one chromosome pair, confirming that our assembly unambiguously distinguished homoeologous chromosomes (Fig. 2a, Supplementary Fig. 4). Centromeric regions contained a tandem repeat with a 314 bp long unit, which was found in all fonio chromosomes (Supplementary Fig. 5). We also re-assembled all the data with the open-source TRITEX pipeline^27^ and the two assemblies showed a high degree of collinearity (Supplementary Fig. 6).

**Table 1:** Statistics of the fonio genome assembly and annotation.

	CM05836
*Length of DeNovoMAGIC3 assembly (Mb)*	*701.662*
Number of scaffolds	8457
N50 (Mb)	10.741
N90 (Mb)	1.009
*Length of genome assembly (Mb)*	*716.471*^a^
*Total length of pseudomolecules (Mb)*	*655.723*
Number of anchored contigs	18,026
N50 of anchored contigs (kb)	83.702
Gap size (Mb)	17.001 (2.6%)
Number of genes	57,021
*Total length of unanchored chromosomes (Mp)*	*60.748*
Number of unanchored contigs	11,129
N50 of unanchored contigs (kb)	9.191
Gap size (Mb)	2.962 (4.9%)
Number of genes	2821
*BUSCO*	
Complete (%)	96.1
Duplicated (%)	84.3
Fragmented (%)	1
Missing (%)	2.9

^a^DeNovoMAGIC3 + Hi-C + optical map.

We compared the fonio pseudomolecule structure to foxtail millet (*Setaria italica*; 2*n* = 2× = 18), a diploid relative with a fully sequenced genome^28^. The fonio genome shows a syntenic relationship with the genome of foxtail millet, with two homoeologous sets of nine fonio chromosomes (Supplementary Fig. 7). Without a clear diploid ancestor, we could not directly disentangle the two sub-genomes based on genomic information from diploid ancestors^29^. We thus used a genetic structure approach based on full-length long terminal repeat retrotransposons (fl-LTR-RT) as an alternative strategy. A total of 11 fl-LTR-RT families with more than 30 elements were identified and defined as a ‘populations’, allowing us to apply genetic structure analyses that are often used in population genomics^30^. We searched for fl-LTR-RT clusters that only appeared in one of the two homoeologous sub-genomes. Out of the 11 fl-LTR-RT populations analyzed, two allowed us to discriminate the sub-genomes (Fig. 2b, Supplementary Fig. 8). The two families belong to the Gypsy superfamily and dating of insertion time was estimated to be ~1.56 million years ago (MYA) (58 elements, 0.06–4.67 MYA) and ~1.14 MYA (36 elements, 0.39–1.96 MYA), respectively. The two putative sub-genomes were designated A and B and chromosome numbers were assigned based on the synteny with foxtail millet (Supplementary Fig. 7).

Gene annotation was performed using the MAKER pipeline (v3.01.02) with 34.1% of the fonio genome masked as repetitive. Transcript sequences of CM05836 from flag leaves, grains, panicles, and whole above-ground seedlings (Supplementary Table 4) in combination with protein sequences of publicly available plant genomes were used to annotate the CM05836 assembly. This resulted in the annotation of 59,844 protein-coding genes (57,023 on 18 pseudomolecules and 2821 on unanchored chromosome) with an average length of 2.5 kb and an average exon number of 4.6. The analysis of the four CM05836 RNA-seq samples showed that 44,542 protein coding genes (74.3%) were expressed (>0.5 transcripts per million), which is comparable to the annotation of the bread wheat genome (Supplementary Table 5)^31,32^.

### Synteny with other cereals

Whole genome comparative analyses of the CM05836 genome with other grass species were consistent with the previously established phylogenetic relationships of fonio^20^. A comparison of the CM05836 A and B sub-genomes identified a set of 16,514 homoeologous gene pairs that fulfilled the criteria for evolutionary analyses (Fig. 2c and Supplementary Data 1). The estimation of synonymous substitution rates (Ks) among homoeologous gene pairs revealed a divergence time of the two sub-genomes of roughly 3 MYA (Supplementary Fig. 9 and Supplementary Data 1). These results indicate that *D. exilis* is a recent allotetraploid species. A Ks distribution using orthologous genes revealed that fonio diverged from the other members of the Paniceae tribe (broomcorn millet (*Panicum miliaceum*), Hall’s panicgrass (*P. hallii*), and foxtail millet (*S. italica*)) between 14.6 and 16.9 MYA, and from the Andropogoneae tribe (sorghum (*Sorghum bicolor*) and maize (*Z. mays*)) between 21.5 and 26.9 MYA. Bread wheat (*T. aestivum*), goatgrass (*Aegilops tauschii*), barley (*Hordeum vulgare*), rice (*O. sativa*), and purple false brome (*Brachypodium distachyon*) showed a divergence time of 35.3–40 MYA (Fig. 2d). The phylogenetic position of *D. exilis* as a basal taxon of the Paniceae allowed us to reconstruct the hypothetical ancestral genomic state of this tribe (Supplementary Data 2).

The hybridization of two genomes can result in genome instability, extensive gene loss, and reshuffling. As a consequence, one sub-genome may evolve dominancy over the other sub-genome^24,31,33–35^. Using foxtail millet as a reference, the fonio A and B sub-genomes showed similar numbers of orthologous genes: 14,235 and 14,153, respectively. Out of these, 12,541 were retained as duplicates while 1694 and 1612 were specific to the A and B sub-genomes, respectively. The absence of sub-genome dominance was also supported by similar gene expression levels between homoeologous pairs of genes (Mann–Whitney *U* test; *p* = 0.66; Supplementary Fig. 10 and Supplementary Data 3).

### Evolutionary history of fonio and its wild relative

To get an overview of the diversity and evolution of fonio, we selected 166 *D. exilis* accessions originating from Guinea, Mali, Benin, Togo, Burkina Faso, Ghana, and Niger and 17 accessions of the proposed wild tetraploid fonio progenitor *D. longiflora*^21^ from Cameroon, Nigeria, Guinea, Chad, Sudan, Kenya, Gabon, and Congo for whole-genome re-sequencing (Supplementary Table 6). The selection was done from a collection of 641 georeferenced *D. exilis* accessions^36^ with the aim of maximizing diversity based on bioclimatic data and geographic origin. We obtained short-read sequences with an average of 45-fold coverage for *D. exili*s (range 36–61-fold) and 20-fold coverage for *D. longiflora* (range 10–28-fold) (Supplementary Data 4). The average mapping rates of the raw reads to the CM05836 reference assembly were 85% and 68% for *D. exilis* and *D. longiflora*, respectively, with most accessions showing a mapping rate of >80% (Supplementary Data 5). After filtering, 11,046,501 high quality bi-allelic single nucleotide polymorphisms (SNPs) were retained (Supplementary Table 7). Nine *D. exilis* and three *D. longiflora* accessions were discarded based on the amount of missing data. The error rate of variant calling (proportion of segregating sites in a re-sequenced CM05836 sample compared to the reference assembly) was 0.04%, which is comparable to other studies^37^. The re-sequenced CM05836 sample showed the lowest genetic divergence from the reference assembly of all re-sequenced accessions (Supplementary Fig. 11a). The SNPs were evenly distributed across the 18 *D. exilis* chromosomes, with a tendency toward a lower SNP density at the chromosome ends (Supplementary Fig. 11b). Approximately 30.2% of the SNPs were gene-proximal (2 kb upstream or downstream of a coding sequence), 9.5% in introns and 6.2% in exons. Of the exonic SNPs, 354,854 (51.6%) resulted in non-synonymous sequence changes, of which 6727 disrupted the coding sequence (premature stop codon). The remaining 333,296 (48.4%) exonic SNPs represented synonymous variants. Forty-four percent of the total SNPs (4,901,160 SNPs) were rare variants with a minor allele frequency of <0.01 (Supplementary Fig. 12). The vast majority of the rare variants was found in a few very diverse *D. longiflora* accessions. The mean nucleotide diversity (*π*) was 6.19 × 10^−4^ and 3.68 × 10^−3^ for *D. exilis* and *D. longiflora*, respectively. Genome-wide linkage disequilibrium (LD) analyses revealed a faster LD decay in *D. longiflora* (*r*^2^ ~ 0.16 at 70 kb) compared to *D. exilis* (*r*^2^ ~ 0.20 at 70 kb) (Supplementary Fig. 13).

**Fig. 3:**
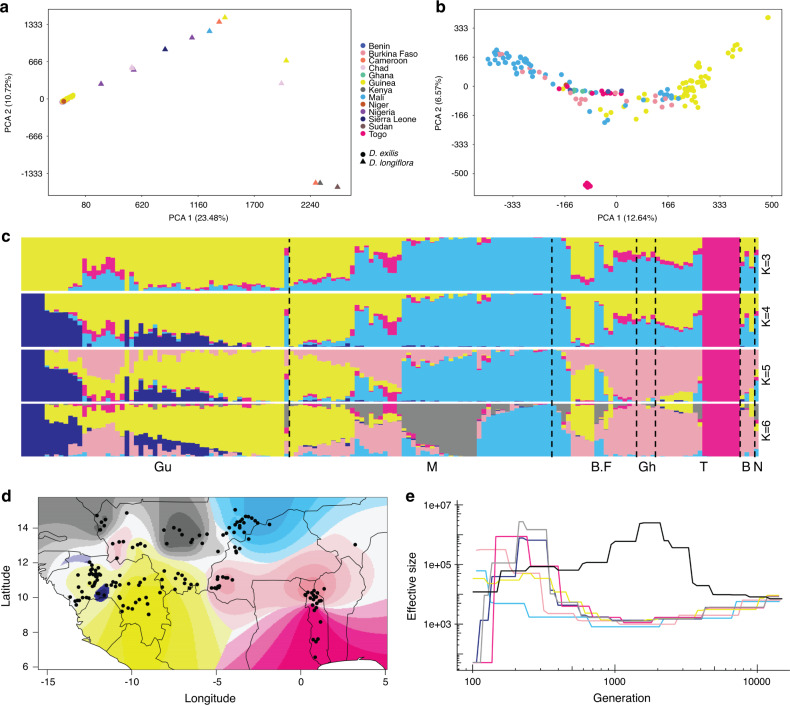
Genetic diversity and structure of *D. exilis* and *D. longiflora* diversity panel. **a** Principal component analysis (PCA) of 157 *D. exilis* and 14 *D. longiflora* accessions using whole-genome single nucleotide polymorphisms (SNPs). *D. exilis* samples (circles), *D. longiflora* (triangles). **b** PCA of *D. exilis* accessions alone. Colors indicate the country of origin. **c** Population structure (from *K* = 3 to *K* = 6) of *D. exilis* accessions estimated with sNMF. Each bar represents an accession and the bars are filled by colors representing the likelihood of membership to each ancestry. Accessions are ordered from west to east; Guinea (Gu), Mali (M), Burkina Faso (B.F), Ghana (Gh), Togo (T), Benin (B), and Niger (N). **d** Geographic distribution of ancestry proportions of *D. exilis* accessions obtained from the structure analysis at *K* = 6. The colors represent the maximal local contribution of an ancestry. Black dots represent the coordinates of *D. exilis* accessions. **e** Effective population size history of *D. exilis* groups based on *K* = 6 and *D. longiflora* (in black).

A PCA showed a clear genetic differentiation between cultivated *D. exilis* and wild *D. longiflora*. The *D. exilis* accessions clustered closely together, while the *D. longiflora* accessions split into three groups (Fig. 3a). The *D. longiflora* group that showed the greatest genetic distance from *D. exilis* contained three accessions originating from Central (Cameroon) and East Africa (South Sudan and Kenya). The geographical projection of the first principal component (which separated wild accessions from the cultivated accessions) revealed that the *D. exilis* accessions genetically closest to *D. longiflora* originated from southern Togo and the west of Guinea (Supplementary Fig. 14a, b).

A PCA with *D. exilis* accessions alone revealed three main clusters. The second principal component separated eight accessions from southern Togo from the remaining accessions (Fig. 3b). In addition, five accessions from Guinea formed a distinct genetic group in the PCA. The remaining accessions were spread along the first axis of the PCA, mainly revealing a grouping by geographic location (Fig. 3b). The genetic clustering was confirmed by genetic structure analyses (Fig. 3c, d). The cross-validation error decreased with increasing *K* and reached a plateau starting from *K* = 6 (Supplementary Fig. 14c). At *K* = 3, the eight South Togo accessions formed a distinct homogenous population. At *K* = 4, the five accessions from Guinea were separated. With increasing *K*, the admixture plot provided some evidence that natural (climate and geography) and human (ethnicity and language) factors had an effect on shaping the genetic population structure of fonio (Fig. 3c, d, Supplementary Fig. 14d). We observed a significant correlation (Pearson’s correlation; *p* < 0.05, df = 155) between the genetic population structure (first principal component of PCA) and climate (i.e., mean temperature and precipitation of the wettest quarters) as well as geography (i.e., latitude, longitude, and altitude) (Fig. 3b, Supplementary Fig. 15, Supplementary Data 6). The relationship between genetic differentiation (genetic distance matrix) and climate was still significant when accounting for geographic distance (partial Mantel test; Mantel *r* = 0.30; *p* = 0.001). A significant correlation was also observed between the genetic distance matrix and the dissimilarity matrices of ethnic and linguistic groups (fisher test; *p* = 0.0005, Mantel test; Mantel *r* ethnic = −0.19; Mantel *r* linguistic = −0.13; *p* = 0.001). The effect of ethnic groups remained significant even when we controlled for geographic and climatic factors (ANCOVA; *p* = 0.045; df = 31) (Supplementary Table 8). Association analyses based on climate variables revealed 38 and 179 loci that might be involved in adaptation to mean temperature and mean precipitation of the wettest quarters, respectively (Supplementary Fig. 16, Supplementary Data 7). Loci associated with temperature contained genes enriched for functions related to hormone metabolic processes, hormone biosynthesis, carbohydrate metabolic processes, homeostatic process, plant development processes, and plant growth (shoot apical meristem maintenance, root growth and development) (Supplementary Data 7). Similarly, genes associated with precipitation were enriched in functions related to plant development and growth (Supplementary Data 7). A genome-wide association study with ethnic groups revealed significant associations for 55 and 227 SNPs with Bambara and Fula ethnic groups, respectively (Supplementary Fig. 17, Supplementary Data 7). In particular, there were three prominent peaks on chromosomes 2A, 2B, and 6A. The peaks on chromosomes 2A and 2B fell into a region that contained a homolog of the Arabidopsis *WSD1* wax ester synthase/diacylglycerol acyltransferase gene^38^. *WSD1* is involved in accumulation of waxes under drought stress, possibly indicating selection for adaptation to drought by certain ethnic groups. Unadmixed populations were mainly found at the geographic extremes of the fonio cultivation area in the north and south, whereas the accessions from the central regions of West Africa tended to show a higher degree of admixture (Supplementary Fig. 18a).

Plotting the spatial distribution of private SNPs (i.e., SNP present only once in a single accession) revealed a hotspot of rare alleles in Togo, Niger, the western part of Guinea, and southern Mali (Supplementary Fig. 18b, c). Rare allele diversity was lower in the eastern part of Guinea, and in southwest Mali. Inference of the *D. exilis* effective population size revealed a decline that started more than 10,000 years ago and reached a minimum between 2000 and 1000 years ago (Fig. 3e). Then, a steep increase of the effective population size occurred to a level that was ~100-fold higher compared to the bottleneck.

### Genomic footprints of selection and domestication

**Fig. 4:**
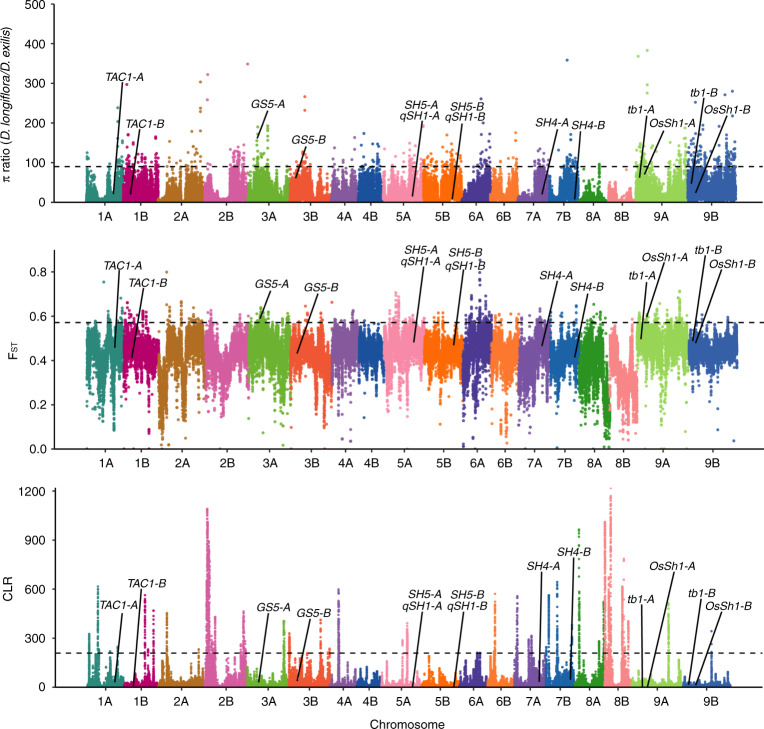
Detection of selection in fonio. Manhattan plots showing detection of selection along the genome based on nucleotide diversity (*π*) ratio, *F*_ST_ and SweeD (from top to bottom). The location of orthologous genes of major seed shattering and plant architecture genes are indicated in the Manhattan plots. The black dashed lines indicate the 1% threshold. Some extreme outliers in the *π* ratio plot are not shown.

**Fig. 5:**
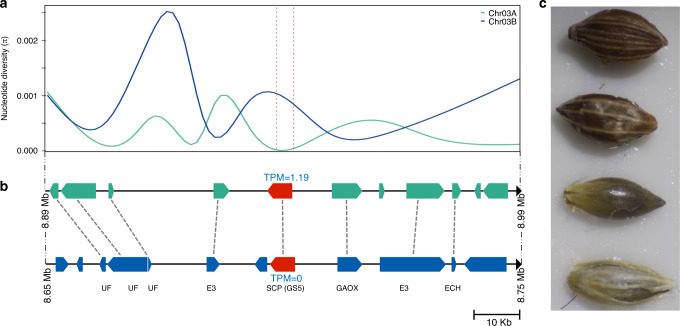
Selective sweep at the *GS5* locus in fonio. **a** Smoothed representation of nucleotide diversity (*π*) in *D. exilis* in a 100 kb window surrounding the ortholog of the rice *GS5* gene. The green curve shows *π* around *DeGS5-3A* on chromosome 3A and the blue curve shows *π* around *DeGS5-3B* on chromosome 3B. The dashed vertical red lines represent the location of the *GS5* genes. The nucleotide diversity was calculated in overlapping 100 bp windows every 25 bp. **b** Schematic representation of the annotated genes in the *GS5* regions and the orthologous gene relationships between chromosomes 3A (green) and 3B (blue). *UF* protein of unknown function, *E3* E3 ubiquitin ligase, *SCP* (*GS5*) serine carboxypeptidase (Dexi3A01G0012320), *GAOX* gibberellin 2-beta-dioxygenase, *ECH* golgi apparatus membrane protein. *DeGS5-3A* and *DeGS5-3B* are indicated in red. The numbers in blue above and below the *GS5* orthologs show their respective expression in transcripts per million (TPM) in grain tissue. **c** Two grains of *D. exilis* (top) and *D. longiflora* (bottom).

We used three complementary approaches to detect genomic regions under selection: (i) a composite likelihood-ratio (CLR) test based on site frequency spectrum (SFS)^39^, (ii) the nucleotide diversity (*π*) ratios between *D. exilis* and *D. longiflora* over sliding genomic windows, and (iii) the genetic differentiation based on *F*_ST_ calculations, again computed over sliding windows. With the CLR test, 78 regions were identified as candidates for signatures of selection. The genetic diversity ratios and *F*_ST_ calculations revealed 311 and 208 regions, respectively (Fig. 4, Supplementary Data 8). We then searched for the presence of orthologs of known domestication genes in the regions under selection. The most striking candidate was one of the two orthologs of the rice grain size *GS5* gene^40^ (Dexi3A01G0012320 referred to as *DeGS5-3A*) that was detected by genetic diversity ratio and *F*_ST_-based calculations (Fig. 4). *GS5* regulates grain width and weight in rice. *D. exilis* showed a dramatic loss of genetic diversity at the *DeGS5-3A* gene (Fig. 5a, b). Domestication of fonio is associated with wider grains of *D. exilis* compared to grains of *D. longiflora* (Fig. 5c). The region of the *GS5* ortholog on chromosome 3B (*DeGS5-3B*) was not identified as being under selection and showed higher levels of nucleotide diversity than the *DeGS5-3A* region (Fig. 5a, b). Only the *DeGS5-3A* but not the *DeGS5-3B* transcript was detected in the *D. exilis* RNA-Seq data from grains. This is in agreement with observations made in rice^40^, where a dominant mutation resulting in increased *GS5* transcript levels affects grain size.

**Fig. 6:**
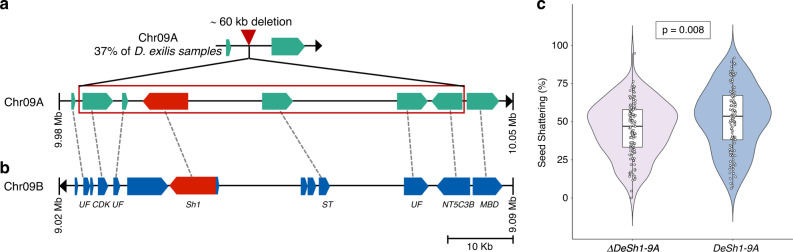
Selective sweep at the *Sh1* locus in fonio. **a** Schematic representation of genes in the orthologous regions of the sorghum *Sh1* gene. The top most panel shows the region on fonio chromosome 9A with the 60 kb deletion as it is found in 37% of all *D. exilis* accessions. The lower panel shows the genes in accessions without the deletion. The *Sh1* ortholog *DeSh1-9A* is shown in red. **b** The *Sh1* ortholog *DeSh1-9B* on chromosome 9B is present in all *D. exilis* accessions. The dashed lines represent orthologous relationships between the two sub-genomes. *UF* protein of unknown function, *CDK* cyclin-dependent kinase, *Sh1* ortholog of sorghum *Shattering 1* (*Sh1*), *ST* sulfate transporter; *NT5C3B* 7-methylguanosine phosphate-specific 5’-nucleotidase; *MBD* methyl-CpG-binding domain. **c** The violin plots show the probability distribution of the seed shattering percentage in fonio accessions carrying the *DeSh1-9A* deletion (∆*DeSh1-9A*) in pink compared to accessions with an intact *DeSh1-9A* in blue. The shape of the distribution indicates that the seed shattering percentages for both groups are concentrated around the median. The center of each plot depicts a boxplot, the box represents the interquartile range (IQR), the middle horizontal line represents the median, the vertical lines going down and up from the box are defined as first quartile (−1.5 IQR) and third quartile (+1.5 IQR), respectively. Circles in the plot represent the seed shattering percentage calculated from individual fonio panicles, one circle per panicle, jitter option was added to show all individual observations that overlapped. Mean seed shattering was 45% in ∆*DeSh1-9A* (±0.18 s.d.) and 52% in *DeSh1-9A* (±0.22 s.d.). (*n* = Three individual panicles of 43 ∆*DeSh1-9A* accessions and 39 *DeSh-9A* accessions, respectively; two-way ANOVA *p* = 0.008; df = 1).

Another domestication gene that was detected in the selection scan was an ortholog of the sorghum *Shattering 1* (*Sh1*) gene^41^. *Sh1* encodes a YABBY transcription factor and the non-shattering phenotype in cultivated sorghum is associated with lower expression levels of *Sh1* (mutations in regulatory regions or introns) or truncated transcripts. Domesticated African rice (*O. glaberrima*) carries a 45 kb deletion at the orthologous *OsSh1* locus compared to its wild relative *O. barthii*^42^. Around 37% of the fonio accessions had a 60 kb deletion similar to *O. glaberrima* that eliminated the *Sh1* ortholog on chromosome 9A (*DeSh1-9A*—Dexi9A01G0015055) (Fig. 6a). This deletion was identified by the lack of mapped reads and was confirmed by PCR amplification and Sanger sequencing. The homoeologous region including *DeSh1-9B* (Dexi9B01G0013485) on chromosome 9B was intact (Fig. 6b). Interestingly, accessions with the *DeSh1-9A* deletion showed a minor (7%) but significant reduction in seed shattering (Fig. 6c) compared to accessions with the intact *DeSh1-9A* gene (ANOVA; *p* = 0.008; df = 1). Accessions carrying this deletion were distributed across the whole range of fonio cultivation, which suggests that the deletion is ancient and might have been selected for in certain regions.

## Discussion

Here, we established a set of genomic resources that allowed us to comprehensively assess the genetic variation found in fonio, a cereal crop that holds great promises for agriculture in marginal environments. The analysis of fl-LTR-RT revealed two sub-genome specific transposon clusters that experienced a peak of activity around 1.1–1.5 MYA, indicating that the two fonio sub-genomes hybridized after this period^43^. The Ks analysis estimated that the two sub-genomes diverged prior to these transposon bursts—3 MYA, suggesting that fonio is an allotetraploid species.

The analysis of effective population size revealed a genetic bottleneck that was most likely associated with human cultivation and domestication. The large increase of effective size for fonio after a period of reduction was most probably due to the development and expansion of fonio cultivation. This expansion appears to be a recent event and occurred during the last millennium. The progression of the effective population size resembles the patterns observed for other domesticated crops, with a protracted period of cultivation followed by a marked bottleneck^44,45^. In the case of fonio, this potential bottleneck appears to be milder compared to other crops. It has been observed that the effective population size of the wild rice (*O. barthii*) from West Africa followed a trend similar to the cultivated African rice (*O. glaberrima*), which has been interpreted as a result of environmental degradation^44^ rather than human selection. In contrast, no signal of population bottleneck was observed for the proposed wild fonio ancestor *D. longiflora*, indicating that the bottleneck observed in fonio is associated with human cultivation. We also highlighted the strong impact of geographic, climatic and anthropogenic factors on shaping the genetic diversity of fonio. Even if fonio is not a dominant crop across West Africa, it benefits from cultural embedding and plays a key role in ritual systems in many African cultures^46^. For example, we observed a striking genetic differentiation between *D. exilis* accessions collected from northern and southern Togo. This can be attributed to both climatic and cultural differences. While the southern regions of Togo receive high annual rainfalls, the northern parts receive <1000 mm annual rainfall and there is a prolonged dry season^47^. Adoukonou-Sagbadja et al.^47^ also noted that there is no seed exchange between farmers of the two regions because of cultural factors.

Despite a genetic bottleneck and the reduction in genetic diversity, fonio still shows many ‘wild’ characteristics such as residual seed shattering, lack of apical dominance, and lodging. We show that orthologs of most of the well-characterized domestication genes from other cereals were not under strong selection in fonio. Interestingly, an ortholog of the rice *GS5* gene (*DeGS5-3A*) was identified in a selective sweep. Dominant mutations in the *GS5* promoter region were associated with higher *GS5* transcript levels and wider and heavier grains in rice^40^. The *DeGS5-3A* gene showed a complete loss of diversity in the coding sequence in *D. exilis*, suggesting a strong artificial selection for larger grains. In contrast, *DeSh1-9A* showed evidence for a partial selective sweep^48^. The non-shattering phenotype associated with the *Sh1* locus in sorghum is recessive^41^ and the deletion of a single *DeSh1* copy only resulted in a quantitative reduction of shattering that might have been selected for in some but not all regions. Whether the 60 kb deletion including *DeSh1-9A* represents standing genetic variation or arose after fonio domestication cannot be determined. The deletion was not identified in any of the 14 re-sequenced *D. longiflora* accessions. Targeting the *DeSh1-9B* locus on chromosome 9B in accessions that carry the *DeSh1-9A* deletion through mutagenesis or genome editing could produce a fonio cultivar with significantly reduced seed shattering, which would form a first important step towards a significant improvement of this crop.

## Methods

### Plant material

The plant material used in this study comprised a collection of 641 *D. exilis* accessions maintained at the French National Research Institute for Development (IRD), Montpellier, France^36,49^. The collection was established from 1977 to 1988. For genome re-sequencing, a panel of 166 accessions (Supplementary Data 6) was selected based on geographic and bioclimatic data. One plant per accession was chosen for DNA extraction and sequencing. Inflorescences of sequenced *D. exilis* plants were covered with bags to prevent outcrossing. Grains of sequenced plants were collected and kept for further analyses. In addition, 17 accessions of *D. longiflora* were collected from specimens stored at the National Museum of Natural History of Paris (Paris, France), IFAN Ch. A. Diop (Dakar, Senegal), National Herbarium of The Netherlands (Leiden, Netherlands), and Cirad (Montpellier, France) (Supplementary Table 6).

### Flow cytometry for genome size estimation

The amount of nuclear DNA in fonio was measured by flow-cytometry^50^. In brief, six different individual plants representing accession CM05836 were measured three times on three different days using a CyFlow® Space flow cytometer (Sysmex Partec GmbH, Görlitz, Germany) equipped with a 532 nm green laser. Soybean (*Glycine max* cultivar Polanka; 2C = 2.5 pg) was used as an internal reference standard. The gain of the instrument was adjusted so that the peak representing G1 nuclei of the standard was positioned approximately on channel 100 on a histogram of relative fluorescence intensity when using a 512-channel scale. The low level threshold was set to channel 20 to eliminate particles with the lowest fluorescent intensity from the histogram. All remaining fluorescent events were recorded with no further gating used. 2C DNA contents (in pg) were calculated from the mean G1 peak (interphase nuclei in G1 phase) positions by applying the formula:

1 $${\mathrm{2C}}\,{\mathrm{nuclear}}\,{\mathrm{DNA}}\,{\mathrm{content}} = \frac{{\left( {{\mathrm{sample}}\,{\mathrm{G1}}\,{\mathrm{peak}}\,{\mathrm{mean}}} \right) \times \left( {{\mathrm{standard}}\,{\mathrm{2C}}\,{\mathrm{DNA}}\,{\mathrm{content}}} \right)}}{{\left( {{\mathrm{standard}}\,{\mathrm{G1}}\,{\mathrm{peak}}\,{\mathrm{mean}}} \right)}}.$$

DNA contents in pg were converted to genome sizes in bp using the conversion factor 1 pg DNA = 0.978 Gb^51^.

### Reference genome sequencing and assembly

High molecular weight (HMW) genomic DNA was isolated from a single CM05836 plant with a dark-treatment of 48 h before harvesting tissue from young leaves. Two paired-end and three mate-pair libraries were constructed with different insert sizes ranging from 450 bp to 10 kb. The 450 bp paired-end library was sequenced on an Illumina Hi-Seq 2500 instrument. The other libraries were sequenced on a Illumina NovaSeq 6000 instrument. In addition, one 10× linked read library was constructed and sequenced with an Illumina NovaSeq 6000 instrument, yielding 75-fold coverage. A de novo whole genome assembly (WGA) was performed with the DeNovoMAGIC3 software (NRGene).

For the super-scaffolding, one Hi-C library was generated using the Dovetail™ Hi-C Library Preparation Kit and sequenced on one lane of an Illumina HiSeq 4000 instrument, yielding ~128-fold coverage. The assembly to Hi-C super-scaffolds was performed with the HiRise^TM^ software v2.0. This generated 18 large super-scaffolds with a size between 25.5 and 49.1 Mb (total size = 642.8 Mb with N50 = 39.9 Mb), while the 19th longest super-scaffold was only 1.5 Mb in size, indicating that the 18 longest super-scaffolds correspond to the 18 *D. exilis* chromosomes.

To generate a BioNano optical map, ultra HMW DNA was isolated using the QIAGEN Genomic-tip 500/G kit (Cat no./ID: 10262) from plants grown in a greenhouse with a dark treatment applied during 48 h prior to collecting leaf tissue. Labeling and staining of the HMW DNA were performed according to the Bionano Prep Direct Label and Stain (DLS) protocol (30206—Bionano Genomics) and then loaded on one Saphyr chip. The optical map was generated using the Bionano Genomics Saphyr System according the Saphyr System User Guide (30247—Bionano Genomics). A total of 1114.6 Gb of data were generated, but only 210.6 Gb of data corresponding to molecules with a size larger than 150 kb were retained. Hybrid scaffolding between the WGA and the optical maps was done with the hybridScaffold pipeline (with the Bionano Access v1.4 default parameters). In total, 39 hybrid scaffolds ranging from 550 kb to 40.9 Mb (total length 657.3 Mb with N50 = 22.6 Mb) were obtained (Supplementary Table 3).

To construct the final chromosome-scale assembly, we integrated the Hi-C super-scaffolds and the hybrid scaffold manually. The 39 hybrid scaffolds generated with the optical map were aligned to the 18 Hi-C super-scaffolds produced by HiRise (Supplementary Table 9). The hybrid scaffolds detected two chimeric DeNovoMAGIC3 scafflods that most likely arose from wrong connections in the putative centromeres. The final chromosome-level assembly was constructed as follows: (i) We used the hybrid assembly to break the two chimeric DeNovoMAGIC3 scaffolds, (ii) merged and oriented the hybrid scaffolds using the Hi-C super scaffolds, and (iii) merged the remaining contigs and scaffolds into an unanchored chromosome.

A second genome assembly was produced using the TRITEX pipeline^27^. Briefly, the initial step using Illumina sequencing data (pre-processing of paired-end and mate-pair reads, unitig assembly, scaffolding and gap closing) was done according the instructions of the TRITEX pipeline [https://tritexassembly.bitbucket.io/]. Integration of 10× and Hi-C reads was done differently. For the 10× scaffolding, we used tigmint v1.1.2^52^ to detect and cut sequences at positions with few spanning molecules, arks v1.0.3^53^ to generate graphs of scaffolds with connection evidence, and LINKS^54^ for a second step of scaffolding. For the Hi-C data, we used BWA^55^ to map the reads against the previous scaffolds and juicer tools v1.5^56^ for the super-scaffolding.

### Preparation of FISH probes and cytogenetic analyses

Libraries of 45 bp long oligomers specific for each fonio pseudomolecule were designed using the Chorus software v1.1^26^ [https://github.com/forrestzhang/Chorus] with the following criteria: -p CGTGGTCGCGTCTCA -l 45 –homology 75 -d 10. The number of oligomers per pseudomolecule was adjusted to ensure uniform fluorescent signals along the entire chromosomes after fluorescence in situ hybridization (FISH). In total, 310,484 oligomers were selected (between 12,647 and 20,000 oligomers per library) and were synthesized by Arbor Biosciences (Ann Arbor, MI, USA). To prepare chromosome painting probes for FISH, the oligomer libraries were labeled directly using 6-FAM or CY3, or indirectly using digoxigenin or biotin. A tandem repeat CL10 with a 314 bp long repetitive unit (155 bp long subunit) was identified after the analysis of fonio DNA repeats with the RepeatExplorer software^57^. A 20 bp oligomer was designed based on the CL10 sequence using the Primer3 software^58^ labeled by CY3 and used for FISH.

Chromosome spreads were prepared from actively growing roots (~1 cm long)^59^. Roots were collected into 50 mM phosphate buffer (pH 7.0) containing 0.2% β-mercaptoethanol, pre-treated in 0.05% 8-hydroxyquinoline for three hours at room temperature, fixed in 3:1 ethanol:acetic acid fixative overnight and stored in 70% ethanol at −20 °C. Chromosome spreads prepared from ~20 roots were washed in distilled water, 1× KCl buffer (pH 4) and root tips were incubated in enzyme mixture containing 4% cellulase and 2% macerozyme for 56 min at 37 °C. The reaction was stopped with TE buffer (pH 7.6), root tips were washed in 100% ethanol, then 30 µl of 9:1 ice-cold acetic acid:methanol mixture was added and root tips were broken with tweezers and dropped onto slide placed in a humid box. The preparations were air-dried, postfixed in 4% (v/v) formaldehyde solution in 2× SSC solution and used for fluorescence in situ hybridization (FISH). FISH was performed with the chromosome painting probes and a probe for the CL10 tandem repeat using a hybridization mixture (30 µl) containing 50% formamide, 10% dextran sulfate in 2× SSC and 10 ng/µl of labeled probe. Hybridization was carried out overnight at 37 °C. Digoxigenin-labeled and biotin-labeled probes were detected using anti-digoxigenin-FITC (Roche Applied Science) and streptavidin-Cy3 (ThermoFisher Scientific/Invitrogen), respectively. The preparations were mounted in Vectashield with DAPI (Vector laboratories, Ltd., Peterborough, UK) to counterstain the chromosomes and the slides were examined with an Axio Imager Z.2 Zeiss microscope (Zeiss, Oberkochen, Germany) equipped with a Cool Cube 1 camera (Metasystems, Altlussheim, Germany) and appropriate optical filters. The capture of fluorescence signals and measure of chromosome lengths were done using the ISIS software 5.4.7 (Metasystems) and final image adjustment was done in Adobe Photoshop CS5.

### Analysis of fl-LTR-RT

fl-LTR-RT were identified using both LTRharvest^60^ (-minlenltr 100 -maxlenltr 40000 -mintsd 4 -maxtsd 6 -motif TGCA -motifmis 1 -similar 85 -vic 10 -seed 20 -seqids yes) and LTR_finder^61^ (-D 40000 -d 100 -L 9000 -l 50 -p 20 -C -M 0.9). Then, the candidate LTRs were filtered using LTR_retriever^62^.

fl-LTRs were classified into families using a clustering approach using MeShClust2^63^ with default parameters and manually curated with dotter^64^ to discard wrongly assigned LTRs. Only LTR-RT families with at least 30 intact copies were considered in genetics analyses, where different families represented ‘populations’ and each fl-LTR-RT copy of a family was considered as an individual. A multiple sequence alignment was performed within each family using Clustal Omega^65^ and the polymorphic sites were identified and converted into variant call format (VCF) using msa2vcf.jar [https://github.com/lindenb/jvarkit]. A principal component analysis (PCA) was performed for each fl-LTR-RT family using the R packages vcfR v.1.8.0^66^ and adegenet v2.1.1^67^. Results were visualized with ggplot2 v3.3.2^68^.

The approximate insertion dates of the fl-LTR-RTs were calculated using the evolutionary distance between two LTR sequences with the formula *T* = *K*/2*μ*, where *K* is the divergence rate approximated by percent identity and *μ* is the neutral mutation rate. The mutation rate used was *μ* = 1.3 × 10^−8^ mutations per bp per year^69^.

### Gene annotation

A combination of homology-based and de novo approaches was used for repeat annotation using the RepeatModeler software [http://www.repeatmasker.org/RepeatModeler/] and the RepeatExplorer software^57^. The results of RepeatModeler, RepeatExplorer and LTR_retriever were merged into a comprehensive de novo repeat library using USEARCH v.11^70^ with default parameters (-id 0.9). The final repeats were classified using the RepeatClassifier module with the NCBI engine and then used to mask the CM05836 assembly.

The protein coding genes were predicted on the masked genome using MAKER (v3.01.02)^71^. Transcriptome reads from flag leaf, grain, panicle and above-ground seedlings were filtered for ribosomal RNA using SortMeRNA v2.1^72^ and for adaptor sequences, quality and length using trimmomatic v0.38^73^. Filtered reads were mapped to the reference sequence using STAR v.2.7.0d^74^ and the alignments were assembled with StringTie v.1.3.5^75^ to further be used as transcript evidence. Protein sequences from *Arabidopsis thaliana*^76^, *S. italica*^28^, *S. bicolor*^77^, *Z. mays*^78^ and *O. sativa*^79^ were compared with BLASTX^80^ to the masked pseudomolecules of CM05836 and alignments were filtered with Exonerate v.2.2.0^81^ to search for the accurately spliced alignments. A de novo prediction was performed using ab initio softwares GeneMark-ES v.3.54^82^, SNAP v.2006-07-28^83^, and Augustus v.2.5.5^84^. Three successive iterations of the pipeline MAKER were performed in order to improve the inference of the gene models and to integrate the final consensus genes. Finally, the putative gene functions were assigned using the UniProt/SwissProt database (Release 2019_08 – [https://www.uniprot.org/]).

### Synteny and comparative genome analysis

To identify syntenic relationships, BLASTP (*E*-value 1 × 10^−5^) was first used to perform all-versus-all protein comparisons between CM05836 and *S. italica*^28^, *Panicum miliaceum*^35^, *Panicum hallii*^85^, *S. bicolor*^77^, *Z. mays*^78^, *O. sativa*^79^, *B. distachyon*^86^, *H. vulgare*^87^, *T. aestivum*^31^ and *A. tauschii*^88^. Then, MCScanX^89^ (-m 25; -s 10) was used for pairwise synteny block detection. For all the comparisons, fonio sub-genomes A and B were analyzed independently and only 1:1 relationships were retained (1:2 relationships for tetraploid broomcorn millet and the ancient tetraploid maize, where individual sub-genomes are not clearly phased).

To estimate the divergence time, we calculated the synonymous substitution rates (*K*s) for each homoeologous and orthologous pair. Briefly, nucleotide sequences were aligned with clustalW^90^ and the *K*s were calculated using the CODEML program in PAML^91^. Then, time of divergence events was calculated using *T* = *K*s/2*λ*, based on the clock-(*λ*) estimated for grasses of 6.5 × 10^−9^ ^92^. We used the maximum likelihood for gene-order analysis (MLGO) tool^93^ to reconstruct the hypothetical ancestral genome of the Paniceae tribe from syntenic‐block data of *D. exilis*, *S. italica*, *P. miliaceum*, and *P. hallii*. Syntenic blocks were identified using MCScanX^89^ using the B sub-genome of *D. exilis* as reference. Then, individual syntenic blocks across all selected species were analyzed according their physical position and orientation in order to combine them into syntenic-block markers^94^ (Supplementary Data 2). Syntenic-block markers along with the known species tree were provided as inputs to the MLGO server to infer the ancestral state of the Paniceae.

To analyze genome dominance, the quantification of transcript abundance and expression were calculated using RSEM v1.3.1^95^ and genes were considered as expressed when expression was >0.5 TPM in at least one tissue. To study the homoeolog expression bias a two-sided Mann–Whitney *U* test was used to examine if the TPM values of sub-genomes A and B differed significantly.

### Re-sequencing of *D. exilis* and *D. longiflora* accessions

One or two young leaves from *D. exilis* or *D. longiflora* seedlings were collected in 2 ml Eppendorf tubes, flash-frozen in liquid nitrogen, and ground with glass beads in a SPEX SamplePrep Geno/Grinder 2010. Samples were mixed with 1.2 ml 2× CTAB buffer (2% CTAB, 200 mM Tris/HCl (pH 8), 20 mM EDTA, 1.4 M NaCl, 1.0% PVP, 0.28 M β-mercaptoethanol) and incubated at 65 °C with periodic mixing for 60 min. Samples were cooled to room temperature and centrifuged at 2000 × *g* for 10 min. Subsequently, 900 μl of the supernatant was transferred to a fresh 2 ml Eppendorf tube and incubated with 800 μl dichloromethane:isoamyl alcohol (24:1) in an overhead-shaker at half-speed at 4 °C for 15 min. After centrifugation at 10,000 × *g* for 15 min, 800 μl of the supernatant was transferred to a fresh 2 ml Eppendorf tube and incubated with 5 μl RNase A (10 mg/ml, EN0531, ThermoFisher Scientific) at 37 °C for 15 min. DNA was precipitated by adding 560 μl isopropanol and mixing the tubes by inversion. Precipitated DNA was pelleted by centrifugation at 4 °C and 10,000 × *g* for 10 min and the supernatant discarded. The DNA pellet was washed in a first ethanol wash (76% ethanol, 200 mM sodium acetate) for 15 min and in a second ethanol wash (76% ethanol, 10 mM ammonium acetate) for 5 min. Subsequently, DNA pellets were air-dried to remove residual ethanol and eluted in 50 μl TE buffer (10 mM Tris/HCl (pH 8), 1 mM EDTA). Extracted DNA was quantified with the Qubit dsDNA HS Assay (Q32851, ThermoFisher Scientific), the purity was confirmed by checking 260/280 and 260/230 ratios on a Nanodrop spectrophotometer, and the integrity was confirmed by analyzing 1 μl per sample on a 1% TAE agarose gel. Library preparation and sequencing were performed by Novogene. Briefly, 1.0 μg DNA per sample was used as input material for the DNA sample preparations. Sequencing libraries were generated using the NEBNext® Ultra II DNA Library Prep Kit following manufacturer’s instructions. Libraries were sequenced using an Illumina NovaSeq 6000 system.

### Mapping of re-sequencing data and variant calling

For quality control of each sample, raw sequence reads were analyzed with the fastqQC tool-v0.11.7 and low-quality reads were filtered with trimmomatic-v0.38^73^ using the following criteria: LEADING:20; TRAILING:20; SLIDINGWINDOW:5:20 and MINLEN:50. The filtered paired-end reads were then aligned for each sample individually against the CM05836 reference assembly using BWA-MEM (v0.7.17-r1188)^55^ followed by sorting and indexing using samtools (v1.6)^96^. Alignment summary, base quality score and insert size metrics were collected and duplicated reads were marked and read groups were assigned using the Picard tools [http://broadinstitute.github.io/picard/]. Variants were identified with GATK v3.8^97^ using the emitRefConfidence function of the HaplotypeCaller algorithm to call SNPs and InDels for each accession followed by a joint genotyping step performed by GenotypeGVCFs. To obtain high confidence variants, we excluded SNPs and InDels with the VariantFiltration function of GATK with the criteria: QD < 2.0; FS > 60.0; MQ < 40.0; MQRankSum < −12.5; ReadPosRankSum < −8.0 and SOR > 4.0. The complete automated pipeline has been compiled and is available on github [https://github.com/IBEXCluster/IBEX-SNPcaller].

A total of 36.5 million variants were called. Raw variants were filtered using GATK v3.8^97,98^ and VCFtools v0.1.17^99^. Variants located on chromosome unanchored, InDels, ‘SNP clusters’ defined as three or more SNPs located within 10 bp, missing data >10%, low and high average SNP depth (14 ≤ DP ≥ 42), and accessions having more than 33% of missing data were discarded. Only biallelic SNPs were retained to perform further analyses representing a final VCF file of 11,046,501 SNPs (Supplementary Table 7). These variants were annotated using snpEff v4.3^100^ with the CM05836 gene models.

### Genetic diversity and population structure

Genetic diversity and population structure analyses were performed using *D. exilis* and *D. longiflora* accessions together, or with *D. exilis* accessions alone. PCA and individual ancestry coefficients estimation were performed using the R package LEA v2.0^101^. The geographical projection of the first PCA axis of the *D. exilis* samples was done and visualized using the Kriging function in the fields v10.3 R package [https://cran.r-project.org/web/packages/fields]^102^. For ancestry coefficients analyses, the snmf function was used with 10 independent runs for each *K* from *K* = 2 to *K* = 10. The optimal *K*, indicating the most likely number of ancestral populations, was determined with the cross-validation error rate. For the two analyses, we considered only SNPs present in at least two accessions (i.e., private SNPs were excluded). We also studied the geographic distribution of private SNPs^103^ (i.e., SNP present only once in a single accession). Genome-wide pairwise LD was estimated independently for *D. exilis* (2,617,322 SNPs) and *D. longiflora* (9,839,152 SNPs). LD decay (*r*^2^) was calculated using the tool PopLDdecay v3.40^104^ in a 500 kb distance and plotted using the ggplot2 v3.3.2 package in R^68^.

### Association of climate, geography, and social factors with the population genetics structure

Natural (i.e., climate, geography) and human (i.e., ethnic and linguistic) factors were tested for an association with the genetic structure of *D. exilis* accessions. Bioclimate data and ethnic and linguistic data were extracted for each of the 166 re-sequenced *D. exilis* accessions from WorldClim v1.4 [https://www.worldclim.org/data/v1.4/worldclim14.html]^105^, passport data (ethnic groups), and Ethnologue v16 (language, [https://www.ethnologue.com/]). The associations between the genetic structure and different factors were evaluated statistically using Pearson’s correlation coefficient test (two-sided) for the bioclimatic and geographic data with the R package stats v3.6.0 (function cor). For the associations of social factors, two-sided Mantel tests (ecodist R package v2.0.5 [https://rdrr.io/cran/ecodist/]) with 999 permutations were performed between the genetic distance matrix of *D. exilis* and the dissimilarity matrix of social factors as inputs. Fisher’s exact tests (stats R package v3.6.0) were performed with the structure data at *K* = 6 and accessions having ancestry thresholds of >70% as input. The *p*-values were computed by Monte Carlo simulation. We used analysis of covariance (ANCOVA) to test for the impact of ethnolinguistic factors on the first PCA coordinates while controlling for other covariates: climatic and geographic variables.

Synthetic climate variables were retrieved from the WorldClim v1.4 database^105^. We focused our analysis on two climate variables that were the most pertinent to annual species, i.e., mean temperature of the wettest quarter (bio8) and mean precipitation of the wettest quarter (bio 16), respectively. A GWAS with these climate variables has been performed^106^. Briefly, we first used the impute method from the R package LEA v2.0^101^ to fill the gaps in the genotypic matrix in order to achieve greater power. We then filtered out SNPs with a MAF below 5%. We used three different models of association to perform the GWAS, efficient mixed-model association (EMMA)^107^ using the R package emma v1.1.2, mixed linear model (MLM) as implemented in the R package gapit v3.0^108^ and latent factor mixed models (LFMM) using LFMM2^109^ as implemented in lfmm R package v2.0. For gapit (MLM), we considered six principal components, which we chose from the previously described structure analysis. The LFMM v2 method implements a Latent Factor Mixed Model that estimates unknown confounding factors (the latent factors) jointly using the genotypic and phenotypic matrices. For this specific model, the estimation of latent factors was performed using both the phenotypic data (i.e., the climate variable in consideration) and a genotypic matrix of SNPs with a MAF higher than 20%. Once the GWAS step was performed, we used a FDR^110^ approach to estimate the significance of the associations for each analysis, retaining a FDR threshold of 5%, resulting in a list of candidate positions for each variable and each GWAS method. We then considered a genomic window of 50 kb upstream and 50 kb downstream for each position as a candidate region, which we denominated quantitative trait locus (QTL). When two QTLs overlapped, they were combined.

To test SNP associations to ethnolinguistic groups, we used the PLINK software v1.90^111^. We filtered out the SNPs with MAF below 5%, more than 10% missing data, and in LD, resulting in 897,796 SNPs. The association of SNPs with each ethnic and linguistic group was tested by a logistic regression model assuming additive genetic effects, using three principal components as covariates to control for population stratification. Significant associations were identified above the threshold of *p* value = 1e^−05^. Manhattan plots were plotted to display the associations using the qqman R package v0.1.4^112^. GO terms for all fonio protein sequences were assigned using the DeepGOPlus model [https://github.com/bio-ontology-research-group/deepgoplus]^113^. We only considered GO labels that have a confident score >0.3. Enrichment analysis for GO biological processes, cellular components, and molecular functions of the genes of interests from climate association analyses was performed using the Fisher exact tests implemented in R package topGO v3.36.0^114^ (algorithm = ‘weight01’). We reported GO terms with enrichment *p* value < 0.05 for each GO-term category.

### Demographic history reconstruction

A Sequentially Markovian Coalescent-based approach^115,116^ was used to infer past evolution of the effective population size of *D. exilis* and *D. longiflora*. This analysis was made with the smc++^117^ software and additional customized scripts [https://github.com/Africrop/fonio_smcpp]. We excluded SNPs that were not usable (e.g. repeated regions) according to msmc-tools advices [https://github.com/stschiff/msmc-tools]. Analysis was done with all nine individuals from the genetically closest group of *D. longiflora* and also with the six groups of *D. exilis* based on the genetic structure. The smc++ vcf2smc tools was used to generate the input file and smc++ inference was performed for the different sets of genotypes using the smc++ cv command, considering 200 and 25,000 generations lower and upper time points boundaries, respectively. All other options were set to default. We considered a generation time of one year and a mutation rate of 6.5 × 10^−9^. Different sets of distinguished lineages were considered for the different sets of individuals (all the samples were considered as distinguished). Graphical representation was made using the ggplot2 v3.3.2^68^ package for R.

### Genome scanning for selection signals

Detection of selection was performed independently with three different methods: (1) Weir and Cockerham *F*_ST_; (2) nucleotide diversity ratios (*π*) were calculated between *D. exilis* and *D. longiflora* using VCFtools v0.1.17^99^ with sliding windows of 50 kb every 10 kb; (3) SweeD v3.3.1^39^ was used on *D. exilis* to detect signatures of selective sweeps based on the composite likelihood ratio (CLR) test within non-overlapping intervals of 3000 bp along each chromosome. We grouped CLR peak positions into one common window if two or several peaks were <100 kb apart. Candidate genomic regions were selected for each method based on top 1% values. A list comprising 34 well-characterized domestication genes (Supplementary Data 8) from major crops was selected and compared by BLAST v2.6.0 against gene models and the genome assembly of *D. exilis* accession CM05836. Each putative orthologous gene was validated by local alignment using ClustalW v2.1^90^. We considered the genes as orthologous when ≥ 70% of the coding sequence (CDS) length hit the genomic sequence of *D. exilis* with at least 70% identity. Validated orthologous genes were crossed with genomic regions under selection using bedtools v2.28.0^118^. To assess the effect of ∆*DeSh1* on seeds shattering, fonio panicles with three racemes were collected from mature plants and placed in 15 ml tubes. Individual tubes were shaken using a Geno Grinder tissue homogenizer (SPEX SamplePrep, Metuchen, NJ) at 1350 rpm speed for 20 s and the percentage of shattering was calculated. ANOVA test was performed with the MVApp [https://mvapp.kaust.edu.sa/]^119^. Two specific primer pairs were designed to confirm the 60 kb deletion. The first primer pair amplifies ~1 kb fragment within the *DeSh1-9A* gene (forward primer 5′-GTGACCATGCCAAGCAGGCG-3′; reverse primer 5′-GCTAGCTTGAGGTATTTACGG-3′). The second primer pair was designed in the flanking region of the deletion and was used to amplify ~750 bp fragment in accessions that carry the deletion (forward primer 5′-GCCGTATATAGTTGCCGCATCA-3′; reverse primer 5′-CCTACCGTTAGATCCGTGCGGA-3′).

### Reporting summary

Further information on research design is available in the Nature Research Reporting Summary linked to this article.

## Supplementary information

Supplementary information

Peer Review

Reporting Summary

Description of Additional Supplementary Files

Supplementary Dataset 1

Supplementary Dataset 2

Supplementary Dataset 3

Supplementary Dataset 4

Supplementary Dataset 5

Supplementary Dataset 6

Supplementary Dataset 7

Supplementary Dataset 8

## Data Availability

Data supporting the findings of this work are available within the paper and its Supplementary Information files. A reporting summary for this Article is available as a Supplementary Information file. The datasets generated and analyzed during the current study are available from the corresponding author upon request. The raw sequencing data used for de novo whole-genome assembly, the raw bionano map, the CM05836 genome assembly, the RNA-seq data for the annotation and the 183 re-sequenced accessions of *D. exilis* and *D. longiflora* for the population genomics analysis are available on EBI-ENA under the study number PRJEB36539 [https://www.ebi.ac.uk/ena/browser/view/PRJEB36539]. The annotation of the CM05836 genome, the Tritex assembly, the probes for the chromosome painting experiment, the gene ontology annotation, the VCF file and *DeSh1* phenotyping data are available on the DRYAD database [10.5061/dryad.2v6wwpzj0]. The plant coding sequences [https://plants.ensembl.org] and [https://genomevolution.org/coge/], the UniProt/SwissProt database (Release 2019_08–[https://www.uniprot.org/]), the embryophyta_odb9 BUSCO dataset [https://busco-archive.ezlab.org/v2/datasets/embryophyta_odb9.tar.gz], the Ethnologue version 16 language [https://www.ethnologue.com/]), and WorldClim version 1.4 [https://www.worldclim.org/data/v1.4/worldclim14.html] databases were downloaded from source for data analyses. Source data are provided with this paper.

## Source data

Source Data

## Code availability

Custom scripts for the SNP calling are available on GitHub: [https://github.com/IBEXCluster/IBEX-SNPcaller]. Custom scripts used to launch the smc++ analysis for fonio genome resequencing data are available on GitHub: [https://github.com/Africrop/fonio_smcpp].
