# Supplementary material for: Fonio millet genome unlocks African orphan crop diversity for agriculture in a changing climate
Source: Nat Commun. 2020 Sep 8;11:4488. doi: 10.1038/s41467-020-18329-4 (PMC7479619; doi:10.1038/s41467-020-18329-4)
Supplement: Supplementary file 1 — Supplementary information [file 41467_2020_18329_MOESM1_ESM.pdf]

Fonio millet genome unlocks African orphan crop diversity for agriculture in a changing climate

Abrouk et al.

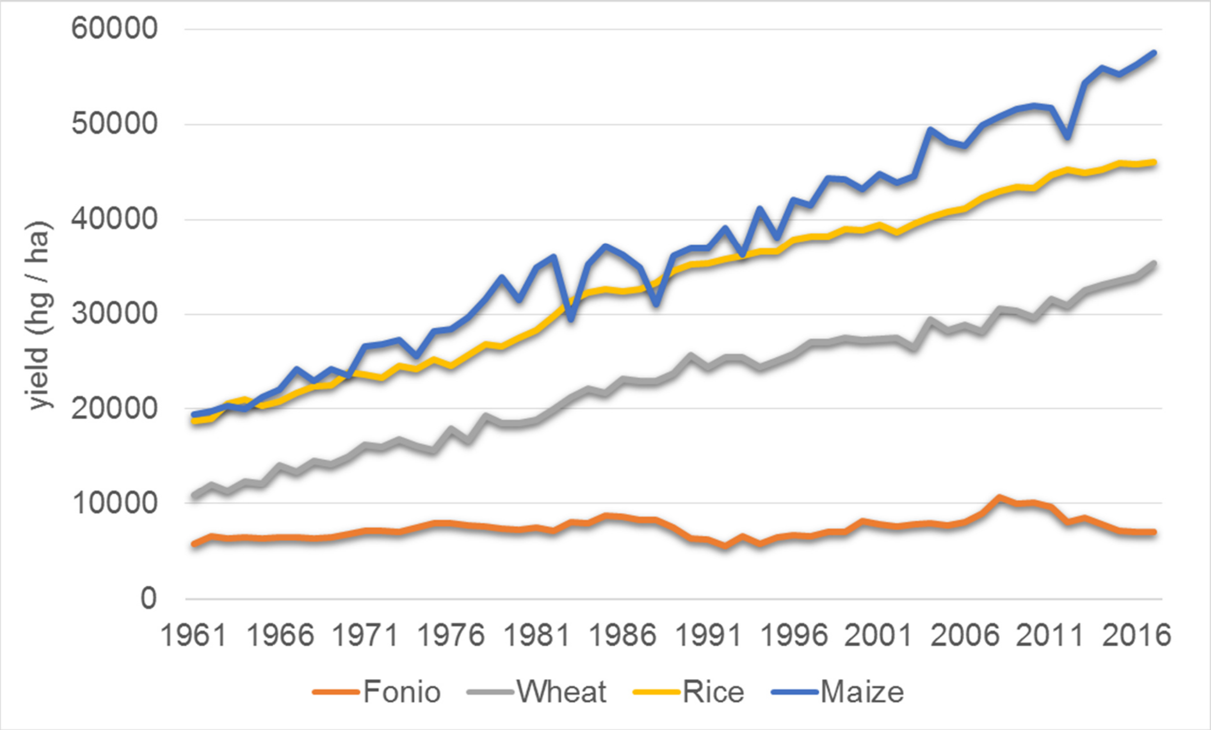

Supplementary Fig. 1. Yields (in hg / ha) of fonio, wheat, rice, and maize from 1961 to 2016. Data were collected from the Food and Agriculture Organization Corporate Statistical Database (FAOSTAT).

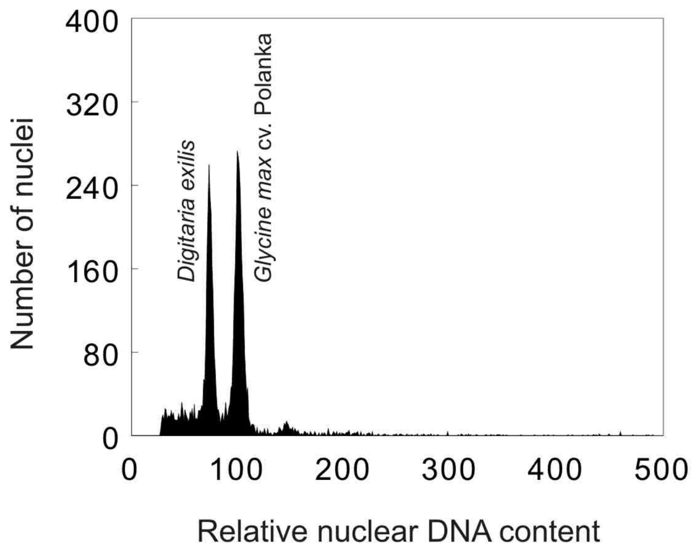

Supplementary Fig. 2. Estimation of nuclear genome size of *Digitaria exilis* using flow cytometry. The histogram of relative nuclear DNA content was obtained after flow cytometric analysis of propidium-iodide stained nuclei of *D. exilis* accession CM05836 and soybean (*Glycine max* cv. Polanka, 2C=2.5 pg), which served as the internal reference standard. Low level threshold was set to channel 20 on a 512-channel scale histogram of relative fluorescence intensity, all remaining fluorescence events were recorded. 2C nuclear DNA content of *D. exilis* was estimated at 1.826 pg (± 0.004 s.d.).

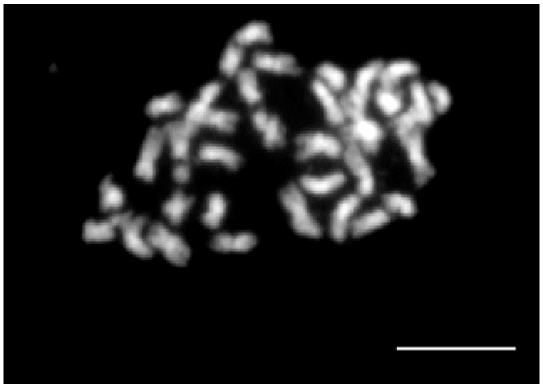

Supplementary Fig. 3. Mitotic metaphase plate of *D. exilis* accession CM05836 (2n = 4x = 36) showing size differences between the chromosomes. The chromosomes were stained by DAPI and the image is shown in white pseudocolor. Five mitotic metaphase plates per slide were captured independently. Scale bar = 5 µm.

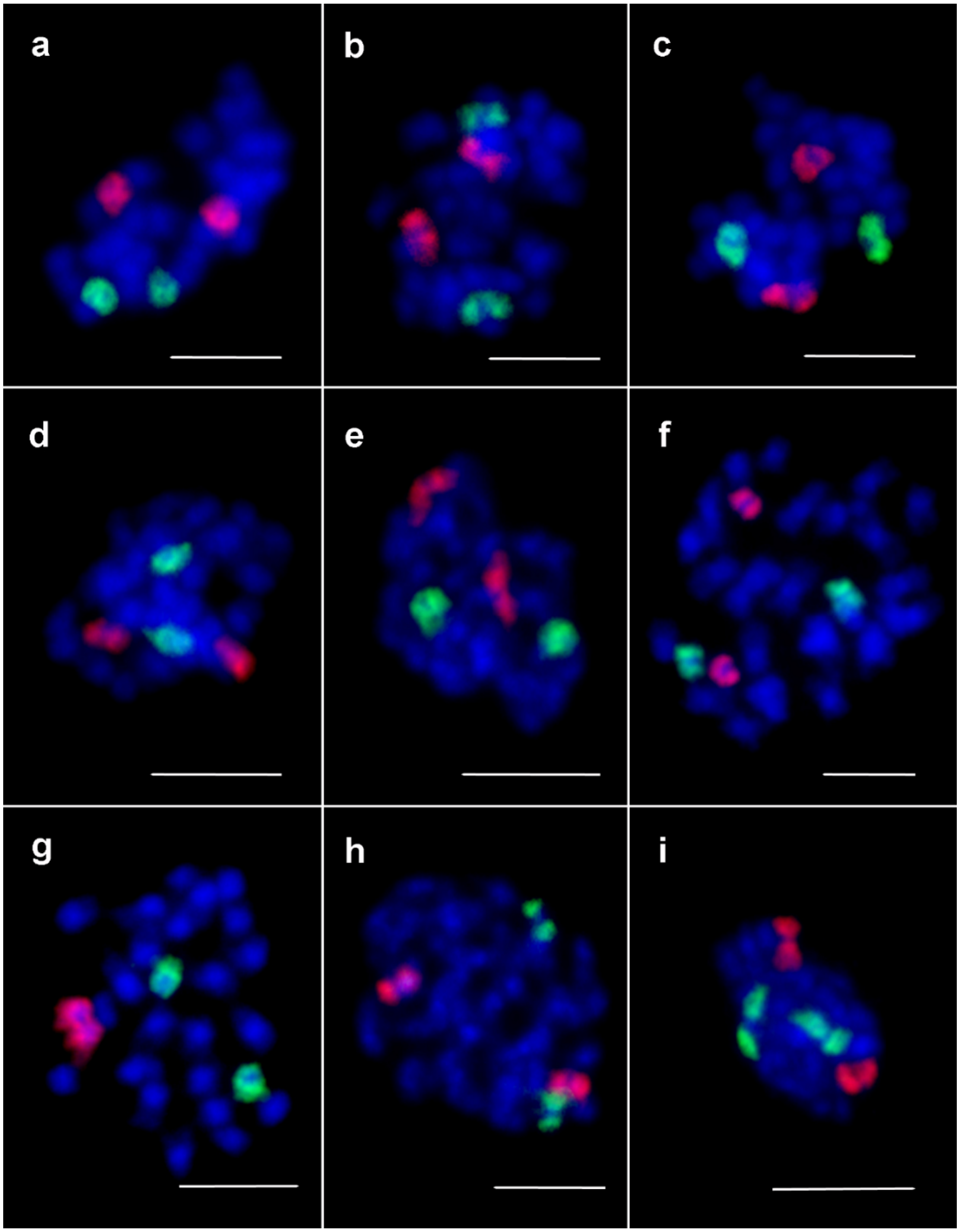

Supplementary Fig. 4. Oligo painting FISH on mitotic metaphase chromosomes of *Digitaria exilis* accession CM05836 (2n = 4x = 36). Each panel shows pairs of homoeologous chromosomes hybridized with 6-FAM (green) or CY3 (red) labeled painting probes. The oligomer probes were designed based on the assembled pseudomolecule sequences: **a,** pseudomolecules 1A (green) and 1B (red); **b,** pseudomolecules 2A (green) and 2B (red); **c,** pseudomolecules 3A (green) and 3B (red); **d,** pseudomolecules 4A (green) and 4B (red); **e,** pseudomolecules 5A (green) and 5B (red); **f,** pseudomolecules 6A (green) and 6B (red); **g,** pseudomolecules 7A (green) and 7B (red); **h,** pseudomolecules 8A (green) and 8B (red); and **i,** pseudomolecules 9A (green) and 9B (red). Chromosomes were counterstained with DAPI (blue). Scale bar = 5 µm. Each oligo painting FISH experiment was repeated independently at least two times.

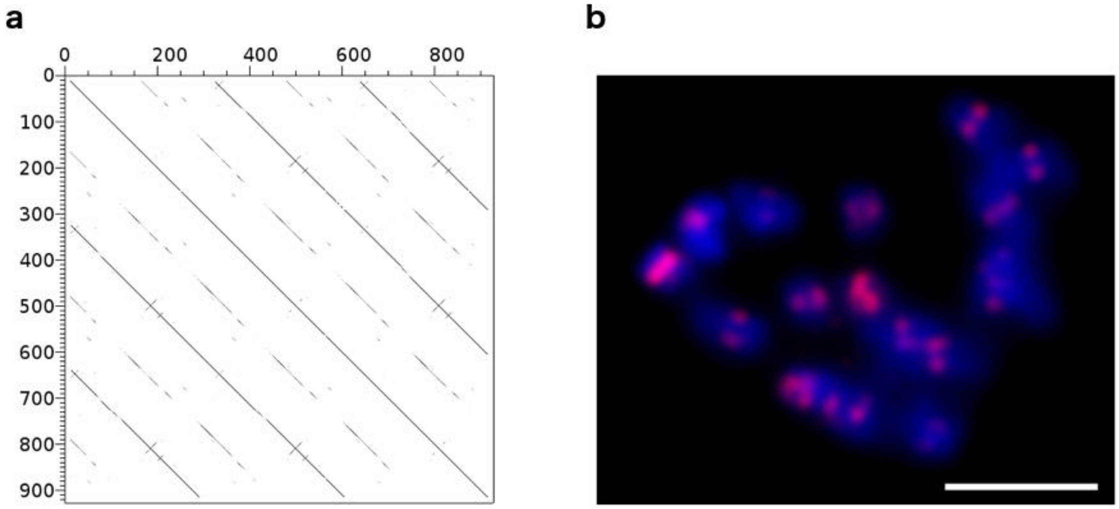

Supplementary Fig. 5. Identification of a centromeric repeat of *Digitaria exilis*. **a,** Dot plot analysis of tandem repeat CL10. **b,** FISH with a CY3 (red)- labeled probe for CL10 on mitotic metaphase plate revealed preferential localization to centromeric regions of all chromosomes. The chromosomes were counterstained with DAPI (blue). Scale bar = 5 µm. The experiment has been repeated independently three times. Source data are provided as a Source Data file.

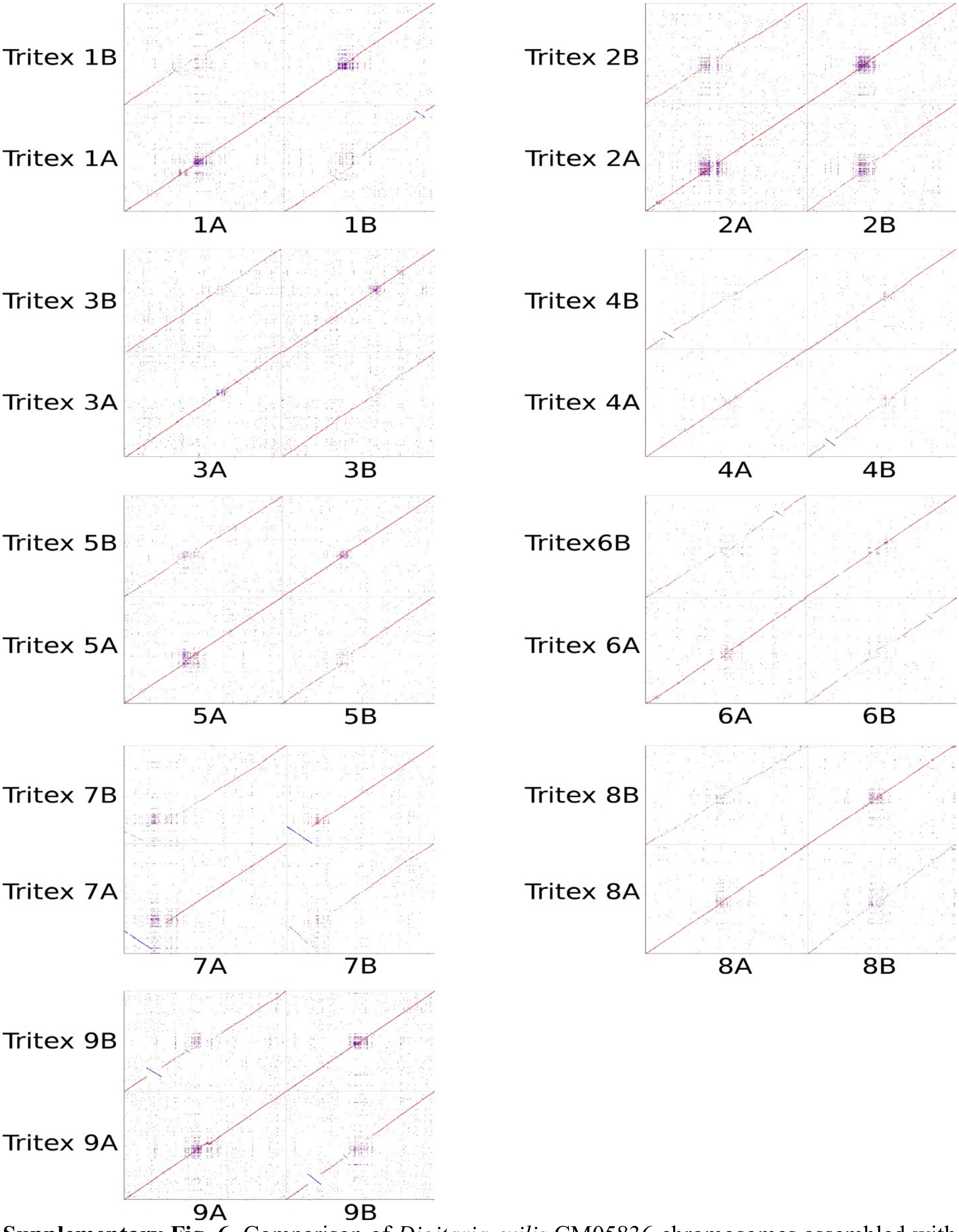

Supplementary Fig. 6. Comparison of *Digitaria exilis* CM05836 chromosomes assembled with DeNovoMAGIC3 and Tritex.

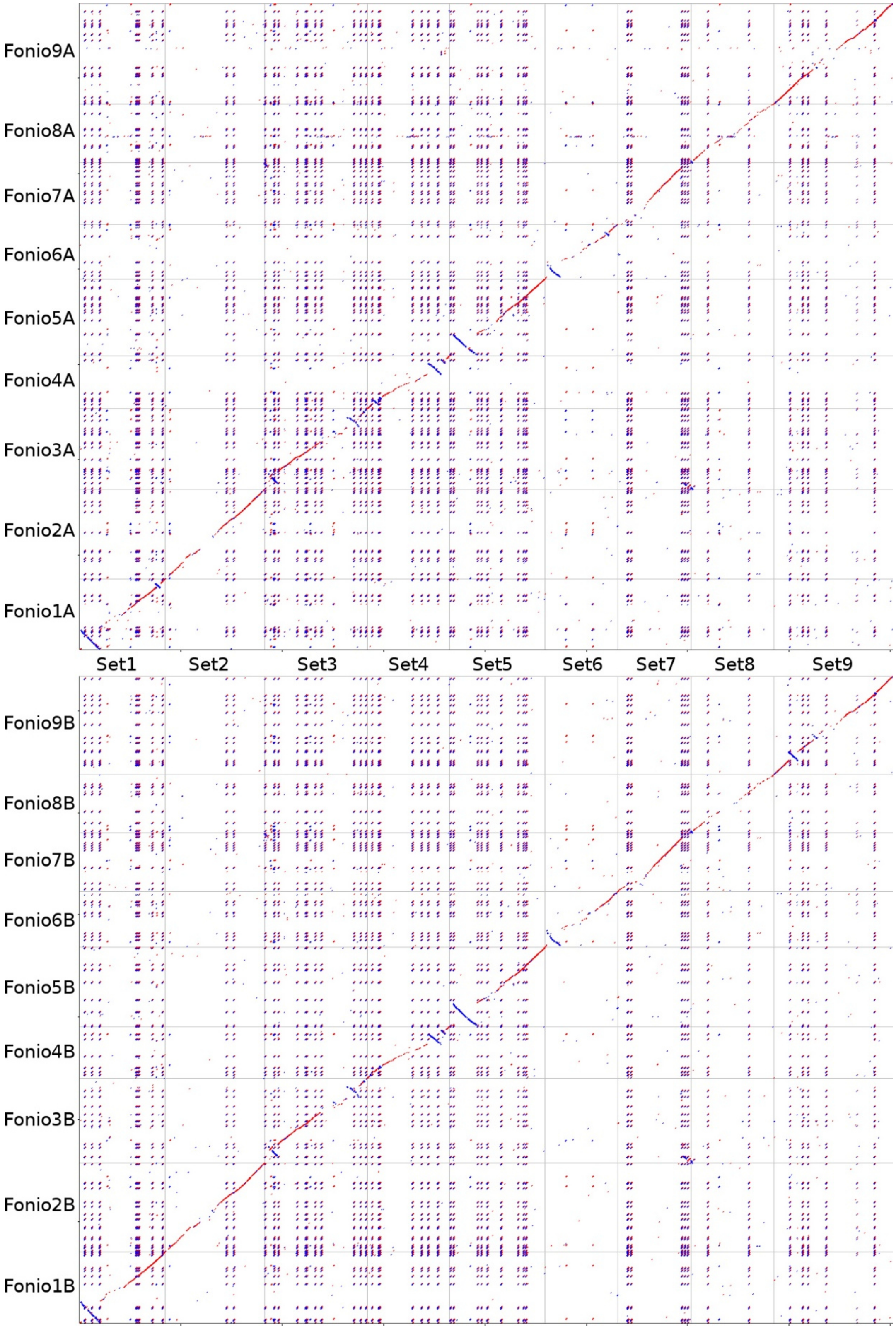

Supplementary Fig. 7. Comparison of *Digitaria exilis* CM05836 sub-genomes against *Setaria italica* (Set) shows syntenic relationship between the two species.

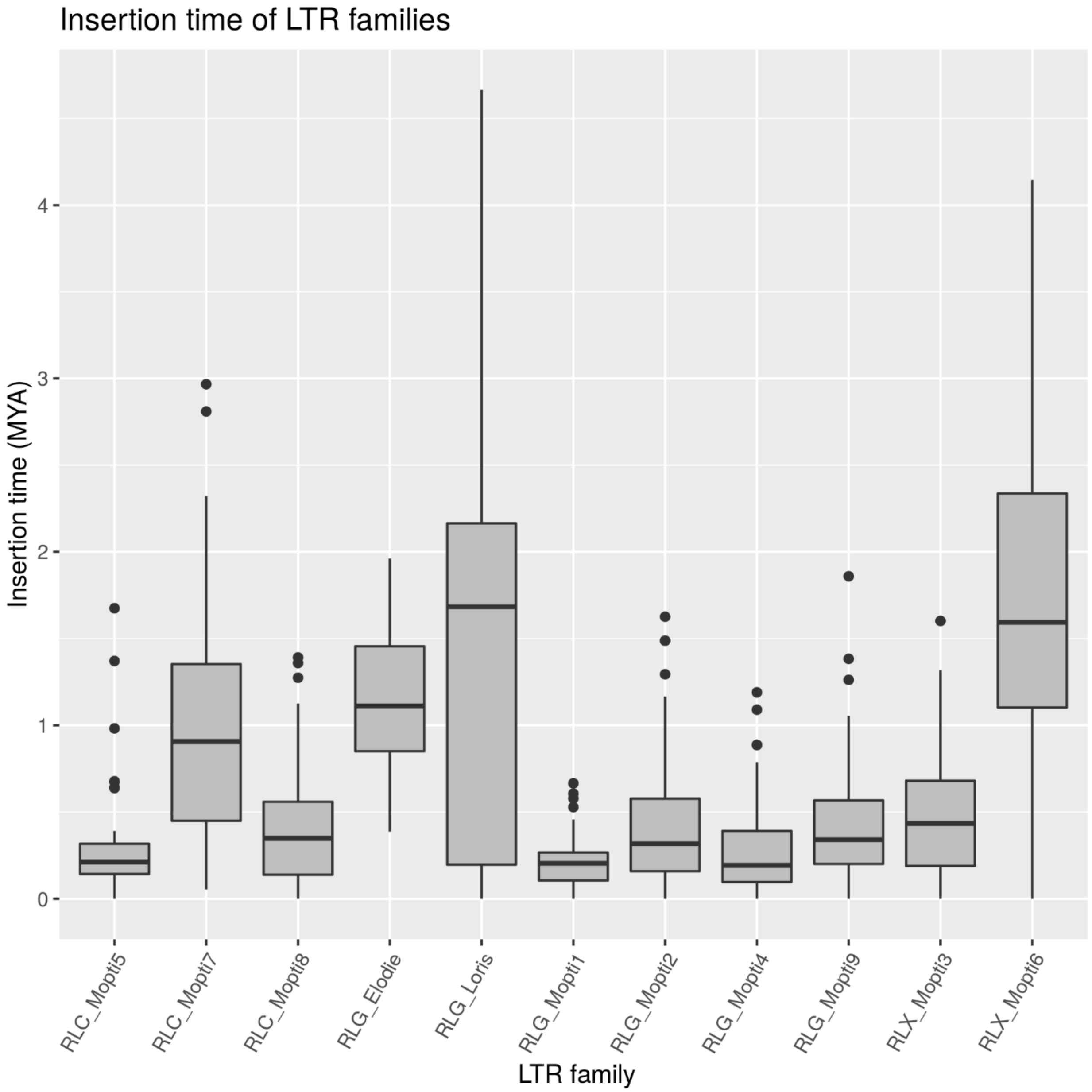

Supplementary Fig. 8. Insertion times of 11 full-length long terminal repeat retrotransposon (fl-LTR-RT) families in *Digitaria exilis* CM05836. Box plots of insertion times (million years ago MYA) for the three Copia families (RLC_Mopti5 (n =38); RLC_Mopti7 (n =31); RLC_Mopti8 (n =60)), six Gypsy families (RLG_Elodie (n =36); RLG_Loris (n =58); RLG_Mopti1 (n =51); RLG_Mopti2 (n =82); RLG_Mopti4 (n =139); RLG_Mopti9 (n =120)) and two unknown families (RLX_Mopti3 (n =52); RLX_Mopti6 (n =31)) are shown. The approximate insertion dates were calculated using the evolutionary distance between two LTR sequences of the same fl-LTR-RT. Clusters RLG_Loris and RLG_Elodie showed a differentiation between the two sub-genomes. Box boundaries indicate the first and third quartiles and the middle line indicate the median of the insertion time. The upper whiskers extend to 1.5 times the interquartile range. Source data are provided as a Source Data file.

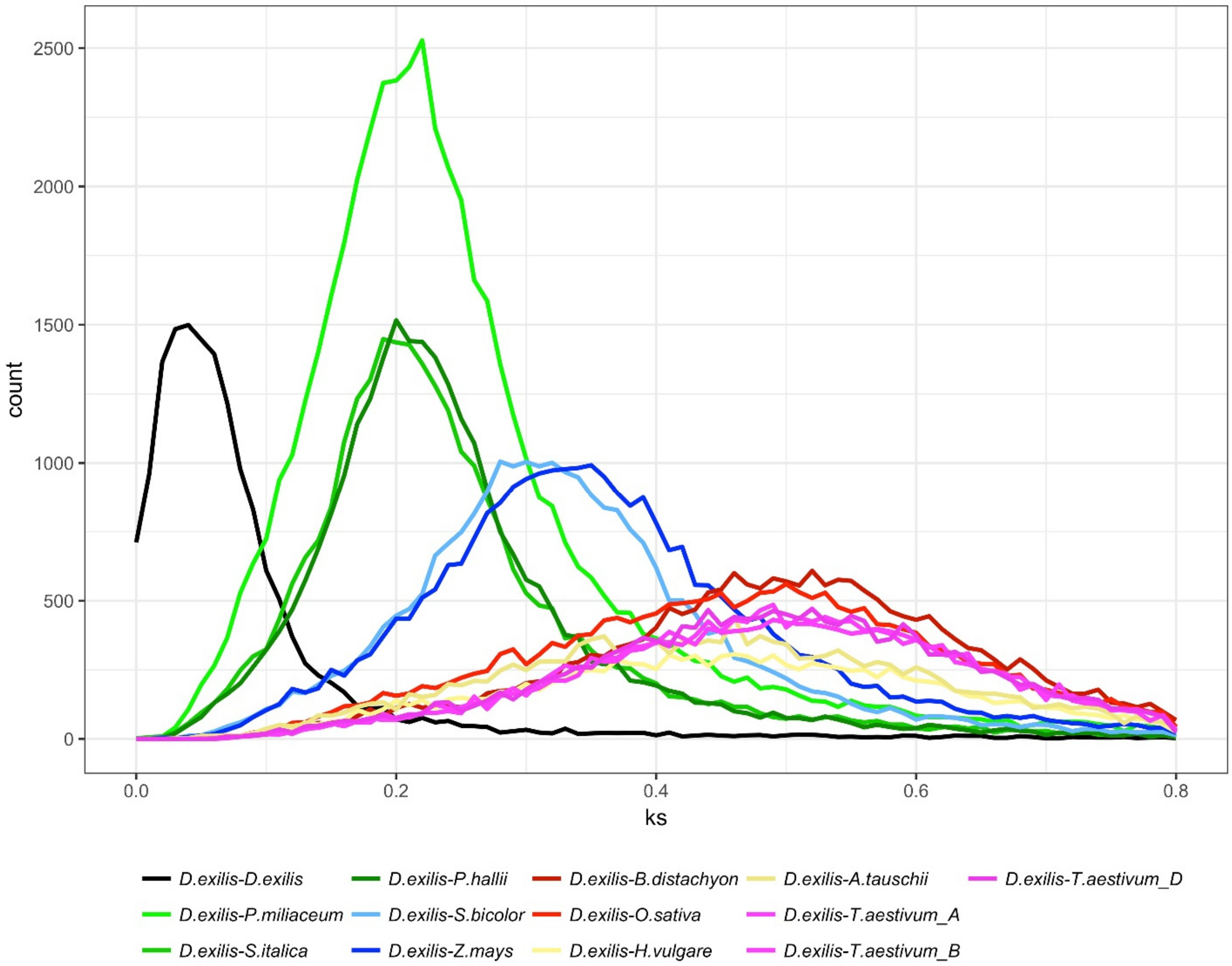

Supplementary Fig. 9. Ks distribution of homoeologous genes within *Digitaria exilis* and orthologous genes between *Digitaria exilis* and other monocots. Time of divergence events were calculated using the formula T = Ks/2λ, based on the clock-(λ) estimated for grasses of 6.5×10^{-9}.

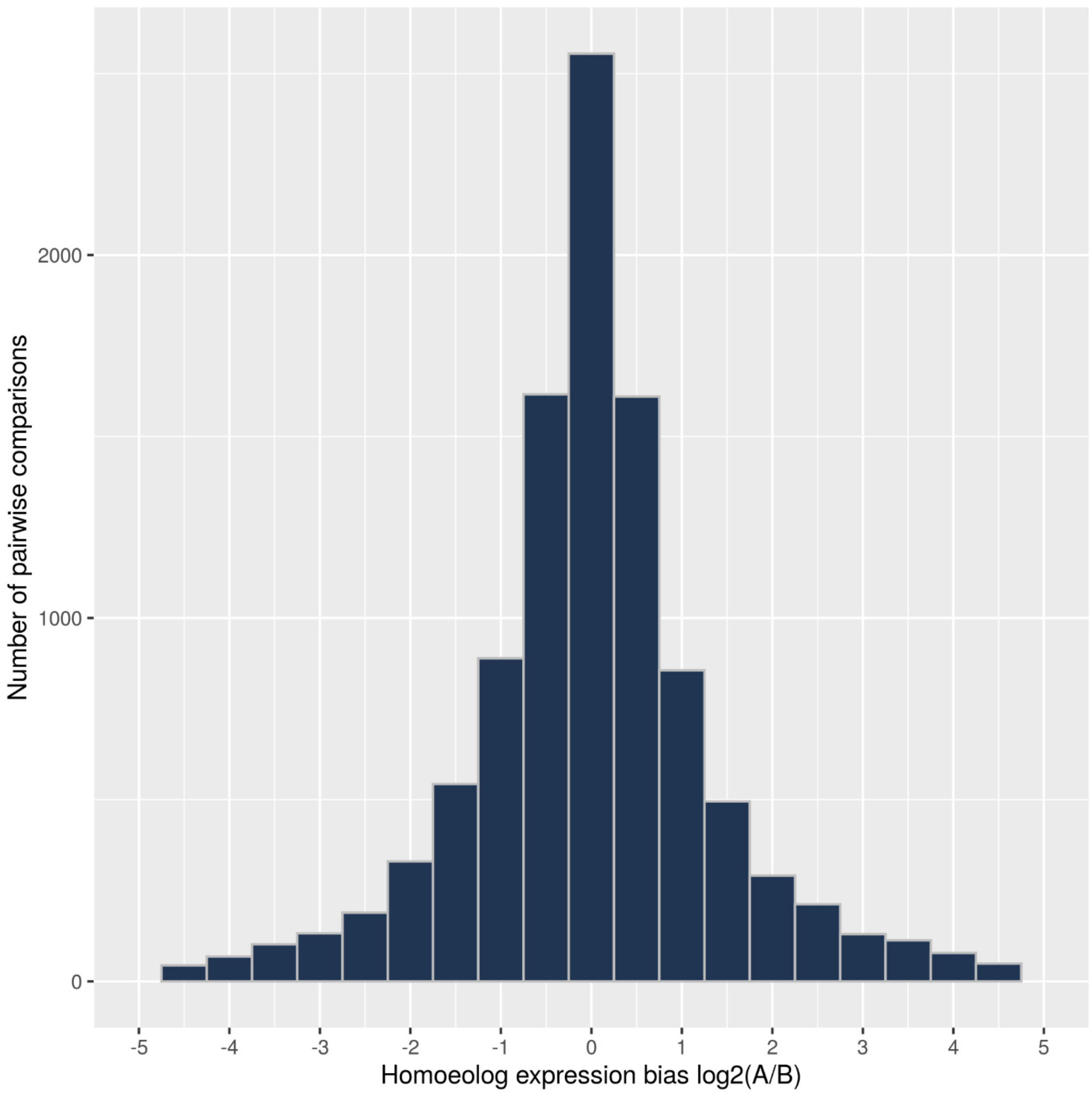

Supplementary Fig. 10. Homoeolog expression bias in *D. exilis* A and B sub-genomes considering all homoeologous gene pairs in flag-leaf, panicle, seedling and grain tissues. Values >0 indicate a bias toward the A sub-genome while values <0 indicate a bias toward the B sub-genome. The p values of two-sided Mann-Whitney U test: flag leaf = 0.96; panicle = 0.59; seedling = 0.73; grains = 0.87.

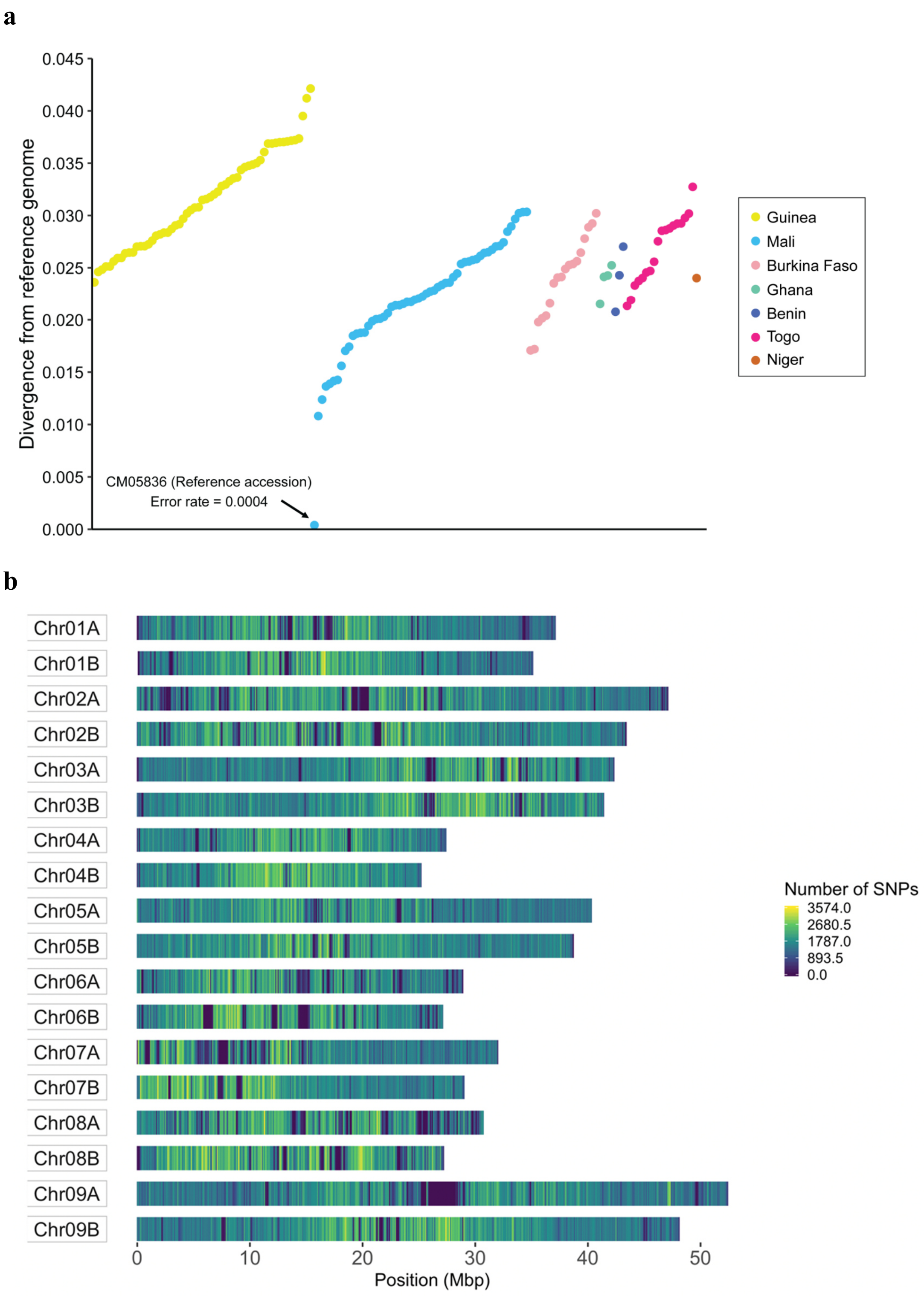

Supplementary Fig. 11. Genetic divergence and SNP distribution. **a,** Genetic divergence of each *D. exilis* accession from the CM05836 reference assembly. Genetic divergence was calculated as the proportion of segregating sites. Colors represent countries of origin. **b,** SNP density across the 18 *D. exilis* chromosomes calculated in a bin sizes of 100 kb.

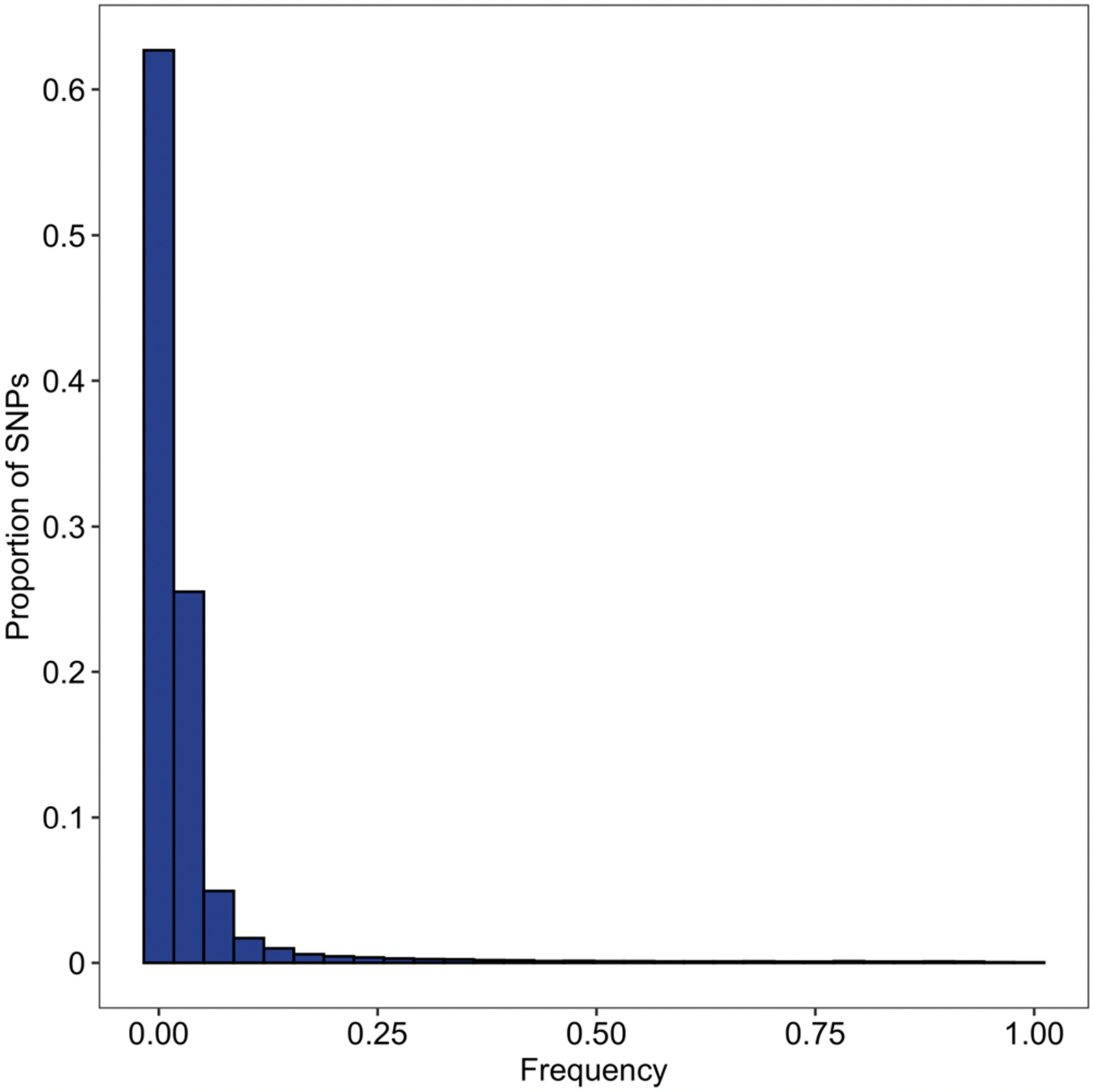

Supplementary Fig. 12. Site frequency spectrum of re-sequenced *D. exilis* and *D. longiflora* accessions.

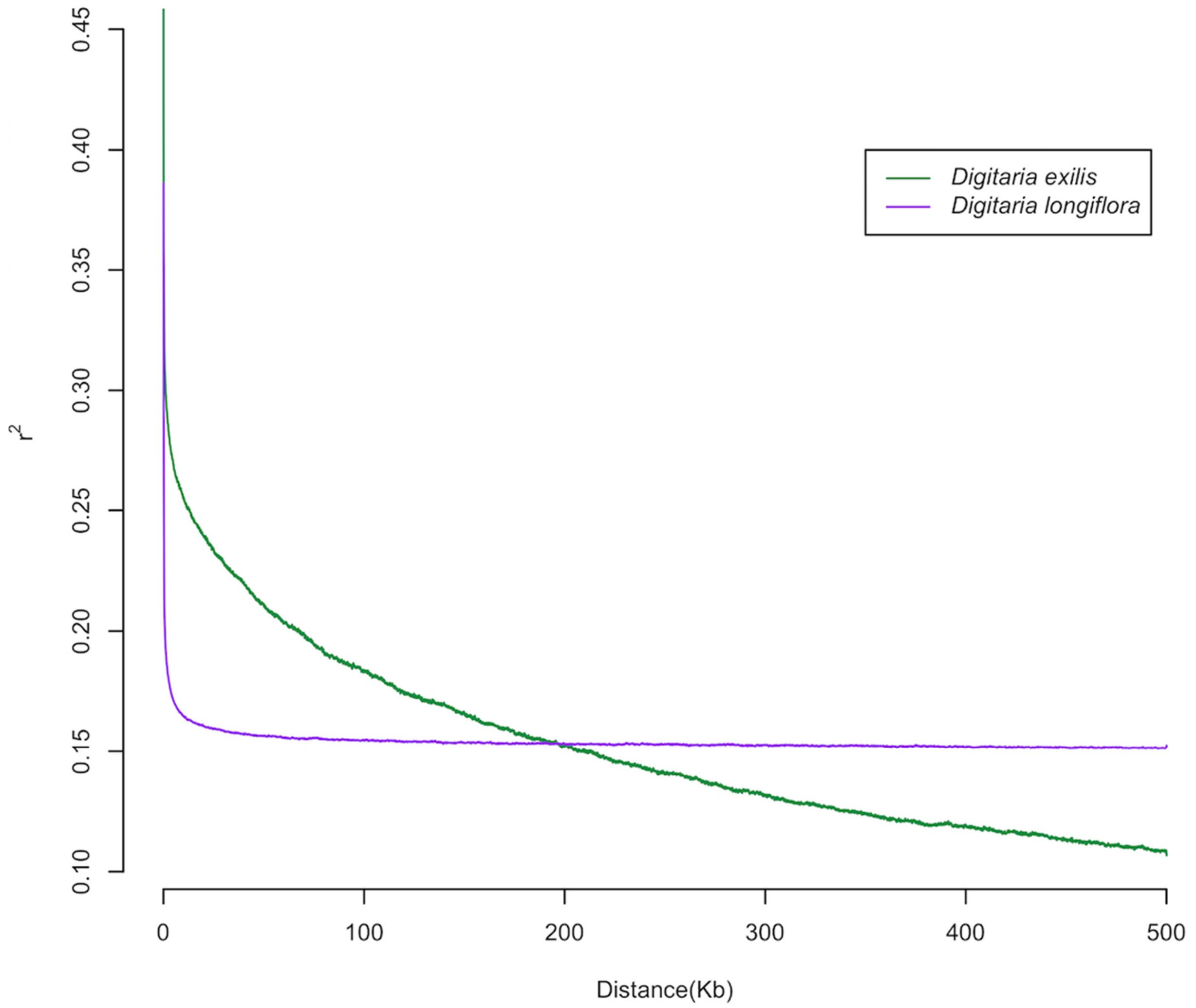

Supplementary Fig. 13. Genome-wide decay of linkage disequilibrium (LD). The difference in LD decay between *D. exilis* and *D. longiflora* at 70 kb +/- 20 kb is statistically significant (two-sample t-test; p = $2.2e^{-16}$; df = 6740, t = -11.515, 95% confidence interval: -0.10703554 and -0.07589332).

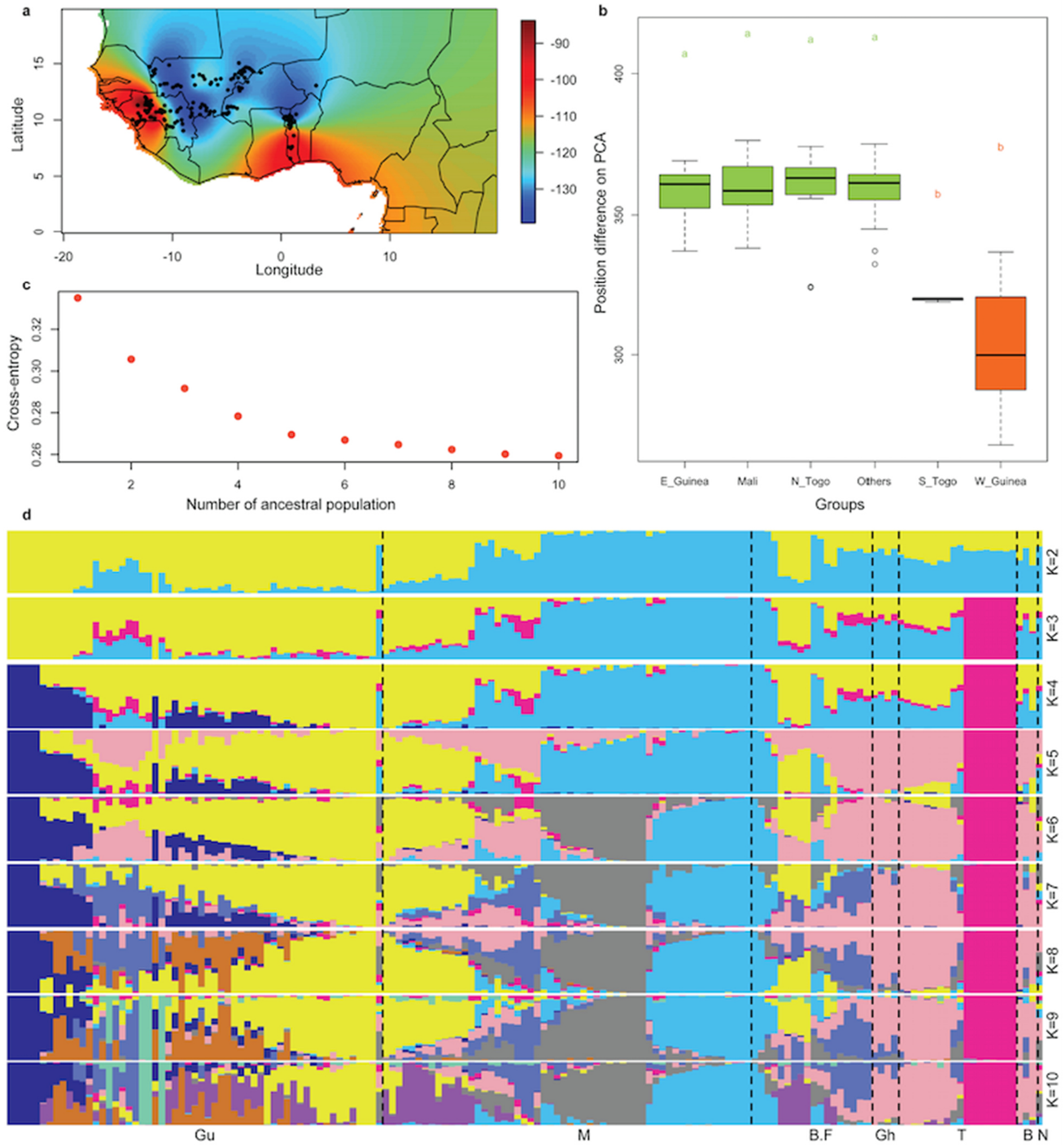

Supplementary Fig. 14. Population structure analyses of fonio. **a**, Geographical projection of the first PCA axis for *D. exilis* accessions. High values (red) indicate higher genetic relatedness of the respective *D. exilis* accessions to *D. longiflora.* Each accession is represented by a black dot. **b**, Box plots showing the differences between the coordinates of *D. exilis* accessions and the closest *D. longiflora* accession (accession L17 from Nigeria). The differences were calculated based on the positions on the first axis of the PCA. Letters indicate groups with a similar distance to *D. longiflora* ($p>0.05$, Tukey HSD two-sided test; df = 151). W_Guinea: western Guinea (n = 26), E_Guinea: eastern Guinea (n = 31), S_Togo: southern Togo (n = 6), N_Togo: northern Togo (n = 12), Mali (n = 56) others: includes accessions from Burkina Faso, Ghana, Benin and Niger (n = 26). Box boundaries indicate the first and third quartile. Lines extending from the boxes (whiskers)

indicate variability outside the lower and upper quartiles (goes from first quartile to the smallest non-outlier point and from third quartile to the largest non-outlier value). The black line in middle of the boxes are the median values for each group. Outliers were plotted as individual points. **c**, Cross-entropy for structure analysis of *D. exilis* samples. The cross entropy was calculated for a number of ancestral populations ranging from *K*=1 to *K*=10. **d**, Population structure (from *K*=2 to *K*=10) of *D. exilis* accessions. Each vertical bar represents an accession and the bars are filled by colors representing the likelihood of membership to each ancestry. The samples are ordered from west to east; Guinea (Gu), Mali (M), Burkina Faso (B.F), Ghana (Gh), Togo (T), Benin (B), and Niger (N).

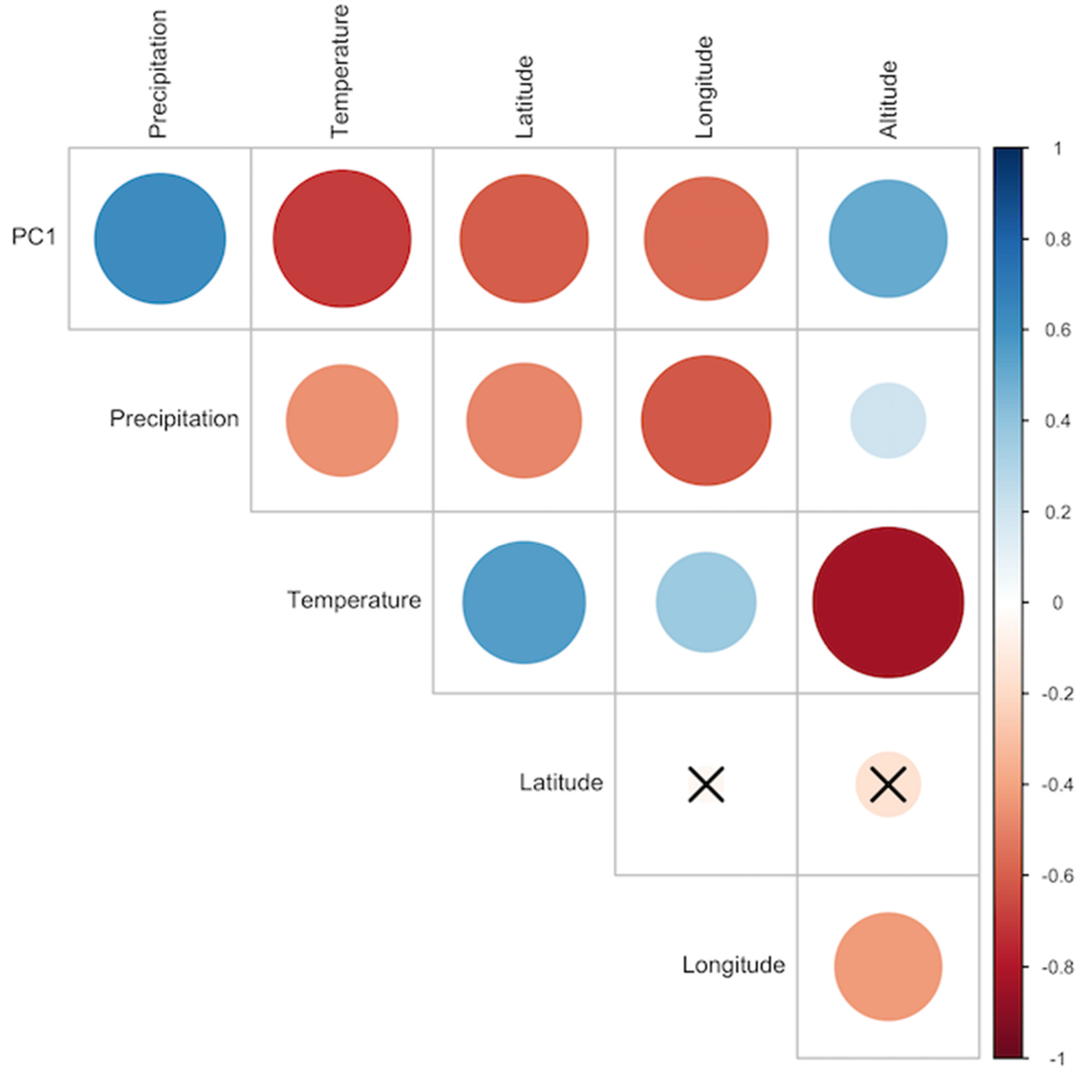

Supplementary Fig. 15. Pearson's correlation coefficients (two-sided) between the first principal coordinate (PC1) and climatic and geographic factors. The sizes of the circles reflect the strength of the correlation. Blue = positive correlation, red = negative correlation. The non-significant correlations, with a p value > 0.05 are indicated with a cross in the individual cells.

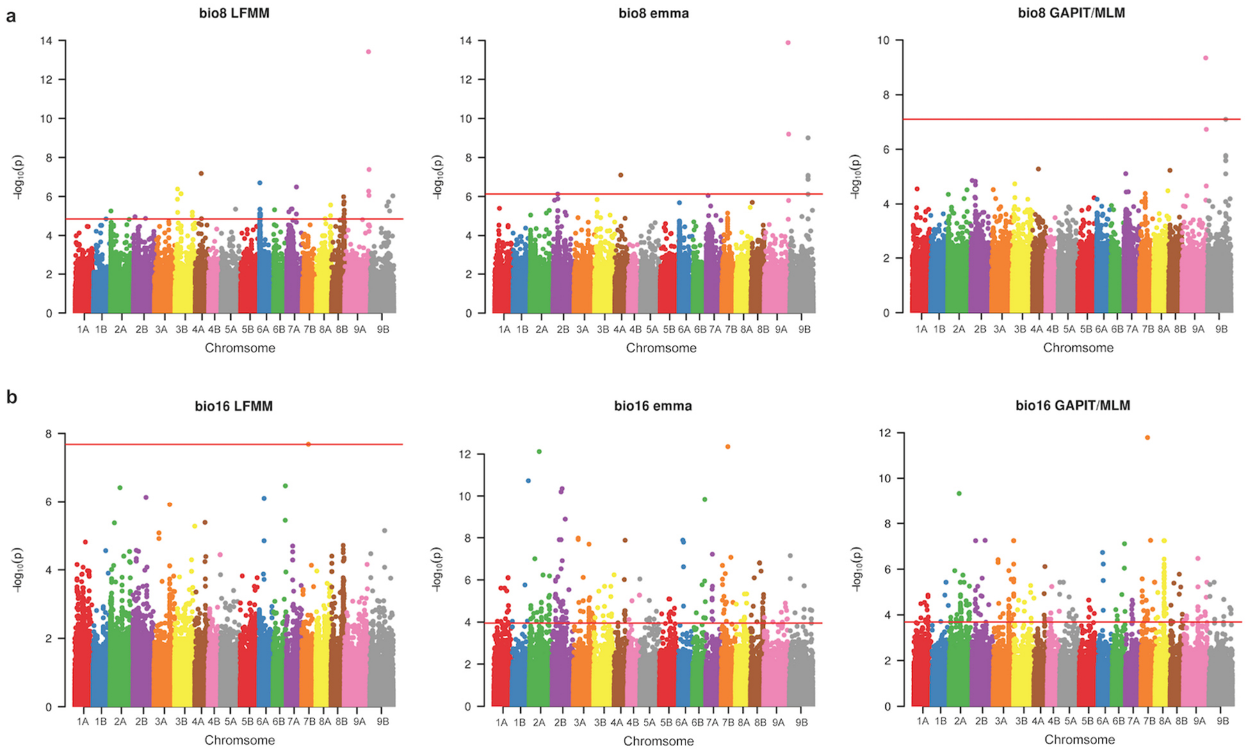

Supplementary Fig. 16. Manhattan plots of the different models preformed for climate association analyses. **a**, Manhattan plots of variable bio8 (the mean temperature of the wettest quarter). **b,** Manhattan plots of variable bio16 (the mean precipitation of the wettest quarter). Different model of association from left to right: LFMM (Latent Factor Mixed Models), EMMA (Efficient Mixed-Model Association), and MLM (Mixed Linear Model). The red horizontal lines represent the FDR thresholds.

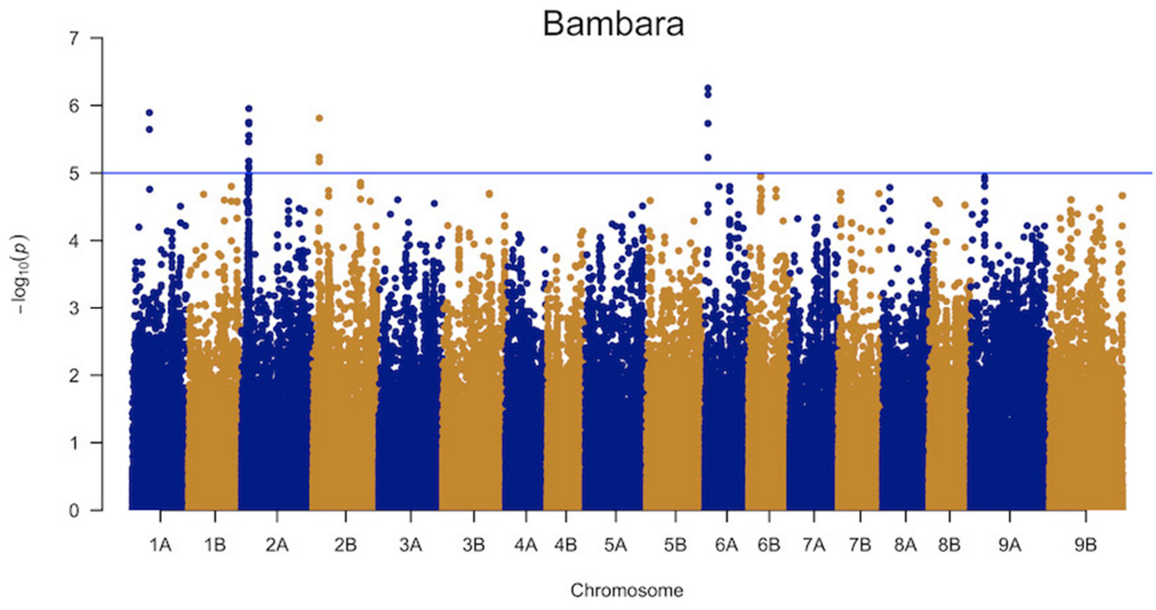

Supplementary Fig. 17. GWAS with different ethnic groups using a logistic regression model. Manhattan plots of ethnic groups (Bambara and Fula) display the association p value for each SNP. The blue horizontal lines represent the P=1e^{-05} significance threshold.

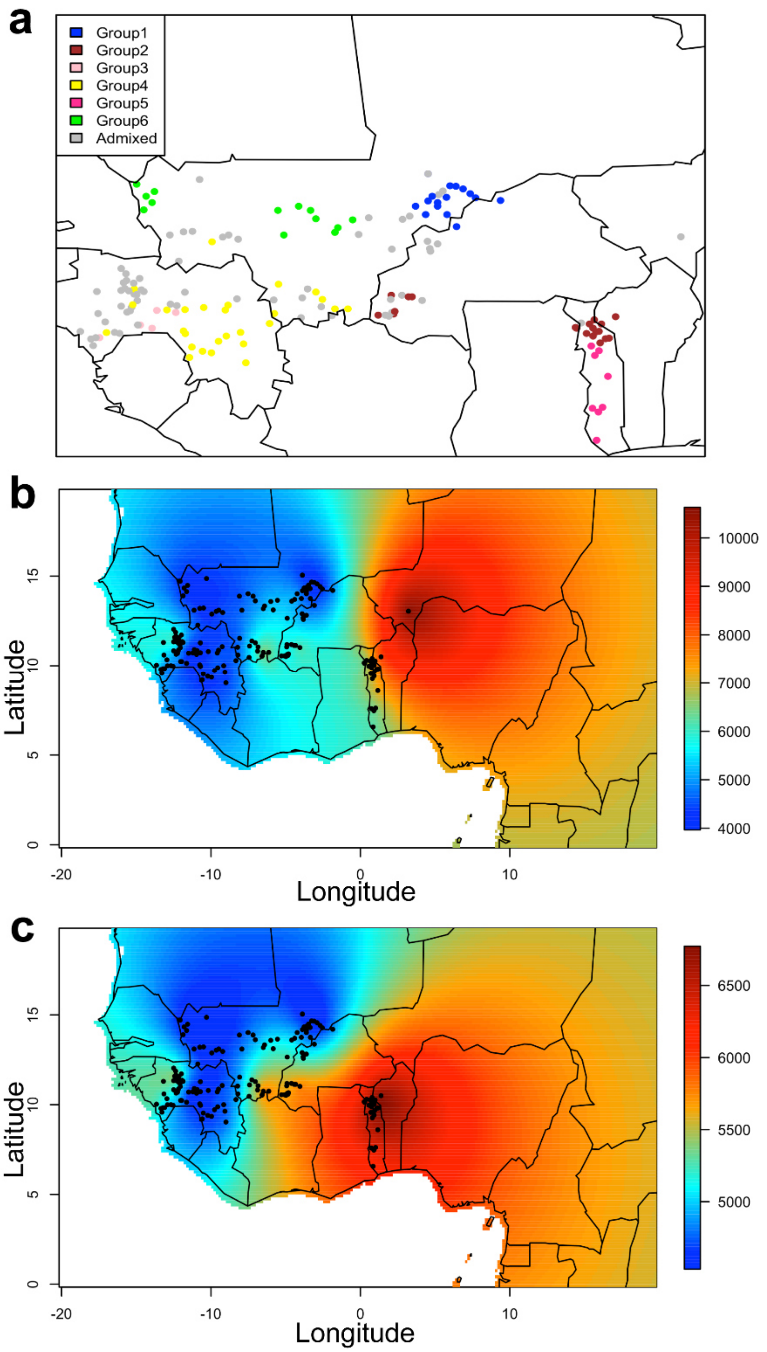

Supplementary Fig. 18. a, Map showing the geographic origin of *D. exilis* accessions colored based on the genetic structure at *K*=6. Gray dots represent the admixed accessions. **b**, and **c**, Geographic distribution of private SNPs (variants found only in one genotype) for all *D.exilis* accessions (**b**), and excluding one accession from Niger (**c**).

Supplementary Table 1. Raw sequencing data information

Library type	Insert size	Approximate depth (coverage)	Total bases (Gb)	ENA sample number
PCR-free PE library (PE250x2)	450-470 bp	197x	176.2	ERS4280600
PCR-free PE library (PE150x2)	700- 800 bp	124x	110.5	ERS4280601
MP (Nextera™ MP Gel Plus)	2-4 kb	89x	79.7	ERS4280599
MP (Nextera™ MP Gel Plus)	5-7 kb	80x	71.6	ERS4280602
MP (Nextera™ MP Gel Plus)	8-10 kb	72x	63.9	ERS4280603
10x Genomics™ Chromium™	N/A	84x	74.6	-
Hi-C	-	122x	109	ERS4280610 – ERS4280605
Bionano	-	235x	210.6	ERZ1300513
RNAseq seedling		-	32.2	ERS4280607
RNAseq grain		-	30.2	ERS4280606
RNAseq flag leaf		-	24.4	ERS4280609
RNAseq panicle		-	24.7	ERS4280608

Supplementary Table 2. Statistics of the CM05836 assembly after Hi-C integration.

Total length of assembly (Mb)	**701.680**
Number of super-scaffolds	**8,293**
Longest super-scaffold (Mb)	**49.06**
N50 (Mb)	**35.296**
N90 (Mb)	**25.563**
Number of gaps	**18,529**
Number of breaks made to input assembly by HiRise	**5**
Number of joints made by HiRise	**174**

Supplementary Table 3. Statistics of the Bionano assembly.

	Bionano Assembly
**Molecules**	
Molecules aligned to NRGene assembly	**739,153**
Filtered molecule N50 (>=150 kb)	**183**
**Assembly**	
Length of Bionano assembly (Mb)	**714.793**
Number of scaffolds	**8,406**
Longest scaffold (Mb)	**40.867**
N50 (Mb)	**21.104**
N90 (Mb)	**6.519**
Length of hybrid scaffold assembly(Mb)	**657.324**
Number of hybrid scaffolds	**39**
Longest hybrid scaffold (Mb)	**40.867**
N50 (Mb)	**22.646**
N90 (Mb)	**11.849**
**Conflict**	
Deletions	**412**
Insertions	**1,858**
Duplications	**5**

Supplementary Table 4. Number of annotated transcripts per tissue and sub-genome.

Tissue	sub-genome A	sub-genome B	unanchored
**flag leaf**	15,073	14,998	1,191
**grain**	13,454	13,447	1,055
**seedling**	18,115	18,064	1,425
**panicle**	19,390	19,430	1,513

Supplementary Table 5. Number of transcripts expressed in multiple CM05836 tissues.

	sub-genome A	sub-genome B	unanchored	Total	
**Never expressed**	7,306	7,046	1,089	15,441	**25.74%**
**Expressed in 1 tissue**	3,231	3,190	312	6,733	**74.26%**
**Expressed in 2 tissues**	2,879	2,875	252	6,006	
**Expressed in 3 tissues**	4,225	4,237	340	8,802	
**Expressed in 4 tissues**	11,092	11,072	837	23,001	

Supplementary Table 6. Origin of wild *D. longiflora* accessions used in this study.

Accession	Country	Collection
4349	NIGERIA	Cirad
67996	GUINEA	Cirad
If-Dlon1	MALI	IFAN Ch. A. Diop
If-Dlon12	SIERRA LEONE	IFAN Ch. A. Diop
L121	SUDAN	National Herbarium of The Netherlands
L17	NIGERIA	National Herbarium of The Netherlands
L3027	CHAD	National Herbarium of The Netherlands
L4148	NIGERIA	National Herbarium of The Netherlands
L8249	GUINEA	National Herbarium of The Netherlands
L9512	CAMEROON	National Herbarium of The Netherlands
L953	KENYA	National Herbarium of The Netherlands
M1070	NIGERIA	Museum of Natural History of Paris
M861	GABON	Museum of Natural History of Paris
M865	CONGO	Museum of Natural History of Paris
M883	CAMEROON	Museum of Natural History of Paris
M905	CHAD	Museum of Natural History of Paris
M949	CHAD	Museum of Natural History of Paris

Supplementary Table 7. Number of SNPs retained after each filtering step.

Filtering step	Number of SNPs retained
1- Initial VCF	36,514,446
2- SNP clusters	24,721,046
3- InDel removal	17,471,641
4- Variant missingness	15,556,597
5- Mean depth	15,378,274
6- Biallelic SNPs	14,076,228
7- Missing data per accession	11,517,157
8- SNPs in unanchored chromosome	11,046,501

Supplementary Table 8. Analysis of covariance (ANCOVA) for testing the effect of all factors together.

Variables	Df	Sum Sq	Mean Sq	F value	Pr(>F)
Temperature	1	4273340	4273340	306.145	< 2.2e-16
Precipitation	1	1138326	1138326	81.5505	7.41E-15
Latitude	1	297866	297866	21.3393	1.07E-05
Longitude	1	1084859	1084859	77.7201	2.26E-14
Altitude	1	1260	1260	0.0903	0.76442
Ethnic	31	683156	22037	1.5788	0.04503
Linguistic	3	64209	21403	1.5333	0.21003

Supplementary Table 9. Alignment of 18 largest Hi-C super-scaffolds (DeNovoMAGIC3 + Hi-C) to the respective hybrid scaffolds (DeNovoMAGIC3 + optical map). The hybrid scaffolds highlighted in red detected chimeras in the original DeNovoMAGIC3 assembly.

Hi-C super- scaffold	Hybrid scaffold
1059	100113 / 444
8292	29 / 106
233	274 / 100007
8290	418 / 528
208	375 / 262
94	100001 / 100018
2	100020 / 100042 / 100043
103	100024 / 100028
8291	100015 / 79
1050	100010 / 528
190	100030 / 103
52	100016 / 248
8293	100004 / 103
86	100045 / 219
8288	138 / 100019
139	8894 / 100051 / 100033
8289	100005 / 100002
117	100003 / 16986
