# Supplementary material for: Fonio millet genome unlocks African orphan crop diversity for agriculture in a changing climate
Source: Nat Commun. 2020 Sep 8;11:4488. doi: 10.1038/s41467-020-18329-4 (PMC7479619; doi:10.1038/s41467-020-18329-4)
Supplement: Supplementary file 3 — Reporting Summary [file 41467_2020_18329_MOESM3_ESM.pdf]

nature research

Corresponding author(s): Krattinger SG

Last updated by author(s): Aug 4, 2020

Reporting Summary

Nature Research wishes to improve the reproducibility of the work that we publish. This form provides structure for consistency and transparency in reporting. For further information on Nature Research policies, see Authors & Referees and the Editorial Policy Checklist .

Statistics

For all statistical analyses, confirm that the following items are present in the figure legend, table legend, main text, or Methods section.

n/a	Confirmed	
☐	☒	The exact sample size (*n*) for each experimental group/condition, given as a discrete number and unit of measurement
☐	☒	A statement on whether measurements were taken from distinct samples or whether the same sample was measured repeatedly
☐	☒	The statistical test(s) used AND whether they are one- or two-sided<br>*Only common tests should be described solely by name; describe more complex techniques in the Methods section.*
☐	☒	A description of all covariates tested
☒	☐	A description of any assumptions or corrections, such as tests of normality and adjustment for multiple comparisons
☐	☒	A full description of the statistical parameters including central tendency (e.g. means) or other basic estimates (e.g. regression coefficient) AND variation (e.g. standard deviation) or associated estimates of uncertainty (e.g. confidence intervals)
☐	☒	For null hypothesis testing, the test statistic (e.g. *F*, *t*, *r*) with confidence intervals, effect sizes, degrees of freedom and *P* value noted<br>*Give P values as exact values whenever suitable.*
☒	☐	For Bayesian analysis, information on the choice of priors and Markov chain Monte Carlo settings
☒	☐	For hierarchical and complex designs, identification of the appropriate level for tests and full reporting of outcomes
☐	☒	Estimates of effect sizes (e.g. Cohen's *d*, Pearson's *r*), indicating how they were calculated

Our web collection on statistics for biologists contains articles on many of the points above.

Software and code

Policy information about availability of computer code

Data collection

Protein coding sequences of Setaria italica, Panicum hallii, Sorghum bicolor, Zea mays, Oryza sativa, Brachypodium distachyon, Hordeum vulgare, Triticum aestivum and Aegilops tauschii were downloaded from EnsemblPlants [https://plants.ensembl.org/index.html] and [https://genomevolution.org/coge/]. Other plant protein were downloaded from UniProt/SwissProt database (Release 2019_08 – [https://www.uniprot.org/]) and the embryophyta_odb9 BUSCO dataset [https://busco-archive.ezlab.org/v2/datasets/embryophyta_odb9.tar.gz].

All biological data have been generated in this study. Bioclimate data have been downloaded from WorldClim v1.4, ethnicity have been collected from passport information, linguistic groups were collected from Ethnologue v16. Known domestication genes were collected from different literature reviews and studies, the references are provided in supplementary data 8

Data analysis

DenovoMAGIC3 software (NRGene) was used for the Whole Genome Assembly

HiRise v2.0 software (Dovetail Genomics) was used for the super-scaffolding

Bionano Access v1.4 was used for the hybrid-scaffolding

Tritex pipeline was used to re-assemble the fonio genome [https://tritexassembly.bitbucket.io/]. The pipeline has been modified for the integration of the 10X sequencing with tigmint v1.1.2, arks v1.0.3 and LINKS. The integration of the Hi-C reads was performed with juicer tools v1.5.

Chorus v1.1 was used to design pseudomolecules probes for the chromosome painting experiment

ltr_finder v1.07, Ltrhavest v1.5.11, LTR_retriever, BEDtools v2.28.0, MeShClust2 v2.3.0, Clustalo v1.2.1, msa2vcf.jar [https://github.com/lindenb/jvarkit], R packages vcfR v.1.8.0 and adegenet v.2.1.1 was used to for identification of the subgenomes

MAKER pipeline v3.01.02 was used for the annotation. It includes RepeatModelersoftware [http://www.repeatmasker.org/RepeatModeler/], RepeatExplorer pipeline, USEARCH v.11 and RepeatMasker4.0.7 for the repeat annotation. SortMeRNA v2.1, trimmomatic v0.38, STAR v.2.7.0d and StringTie v.1.3.5 was used for the transcript assembly. BLASTX and Exonerate v.2.2.0 were used for the comparison with other protein coding genes sequences. GeneMark-ES v.3.54, SNAP v.2006-07-28 and Augustus v.2.5.5 were used for ab initio prediction.

MCScanX and CODEML program were used for the comparative analysis

RSEM v1.3.1 was used for the gene expression analysis

fastQC v-0.11.7 were used to analyze raw sequence reads.
Trimmomatic-v0.38 for low quality reads filtering.
BWA-MEM (v0.7.17-r1188) for aligning reads against reference genome assembly.
The samtools v-1.6 for sorting and indexing.
Picard tools [http://broadinstitute.github.io/picard/] was used to mark duplicated reads and assign group reads.
GATK-v3.8 was used to call for variants and basic filtering.
VCFtools v0.1.17 used to filter VCF file, and produce statistical analyses of population data (i.e., Minor allele frequency, SNP density, nucleotide diversity, and FST calculation..etc).
PopLDdecay v3.40 for LD decay estimation.
The snpEff v-4.3 was used to annotate variants with gene models.
PLINK software v1.90 for ethnic association
DeepGOPlus model [https://github.com/bio-ontology-research-group/deepgoplus] used for assigning GO labels
SMC++ program [https://github.com/stschiff/msmc-tools] used for estimating effective population size
SweeD v-3.3.1 was used to detects elective sweeps.
BLAST v2.6.0 and ClustalW v2.1 were used to identify and validate putative orthologous domestication genes.
The BEDtools v2.28.0 used for crossing orthologous domestication genes with genomic regions under selection.
MVApp [https://mvapp.kaust.edu.sa/] was used for phenotypic analyses and ANOVA test
R packages used:
ggplot2 v3.3.2, vcfR v.1.8.0, adegenet v2.1.1, LEA v2.0, fields v10.3, stats v3.6.0, ecodist v2.05, qqman v0.1.4, topGO v3.36.0, emma v1.1.2 gapit v3.0, LFMM v2.0
Custom R scripts for effective size analysis are available here: [https://github.com/Africrop/fonio_smcpp]
Automated SNP calling pipeline is available here: [https://github.com/IBEXCluster/IBEX-SNPcaller]

For manuscripts utilizing custom algorithms or software that are central to the research but not yet described in published literature, software must be made available to editors/reviewers. We strongly encourage code deposition in a community repository (e.g. GitHub). See the Nature Research guidelines for submitting code & software for further information.

Data

Policy information about availability of data

All manuscripts must include a data availability statement. This statement should provide the following information, where applicable:

- Accession codes, unique identifiers, or web links for publicly available datasets
- A list of figures that have associated raw data
- A description of any restrictions on data availability

The raw sequencing data used for de novo whole-genome assembly, the raw bionano map, the CM05836 genome assembly, the RNA-seq data for the annotation and the 183 re-sequenced accessions of D. exilis and D. longiflora for the population genomics analysis are available on EBI-ENA under the study number PRJEB36539.
The annotation of the CM05836 genome, the Tritex assembly, the probes for the chromosome painting experiment, the gene ontology annotation, the VCF file and DeSh1 phenotyping data are available on the DRYAD database under [https://doi.org/10.5061/dryad.2v6wwpzj0]. The plant coding sequences [https://plants.ensembl.org] and [https://genomevolution.org/coge/], the UniProt/SwissProt database (Release 2019_08 – [https://www.uniprot.org/]), the embryophyta_odb9 BUSCO dataset [https://busco-archive.ezlab.org/v2/datasets/embryophyta_odb9.tar.gz], the Ethnologue version 16 (language, [https://www.ethnologue.com/]) and WorldClim version 1.4 [https://www.worldclim.org/data/v1.4/worldclim14.html] databases were downloaded from source for data analyses.
The source data underlying Figures 2b, 2d, as well as Supplementary Figures 5b, and 8 are provided as a Source Data file.

Field-specific reporting

Please select the one below that is the best fit for your research. If you are not sure, read the appropriate sections before making your selection.

☒ Life sciences ☐ Behavioural & social sciences ☐ Ecological, evolutionary & environmental sciences

For a reference copy of the document with all sections, see nature.com/documents/nr-reporting-summary-flat.pdf

Life sciences study design

All studies must disclose on these points even when the disclosure is negative.

Sample size	Sample size for population analyses for D. exilis was defined based on bioclimate data and geographic location to maximize diversity.<br>In total 157 individuals were analyzed in which they covered the different climate and geography.<br>For DeSh1 phenotyping, three individual panicles of 39 DeSh-9A accessions and 43 ΔDeSh1-9A accessions were defined based on finding comparable panicles across accessions (i.e., similar size, and seed content).
Data exclusions	Private SNPs (i.e., SNP present only once in a single individual) have been excluded from PCA and sNMF (diversity and structure) analyses as it can bias the analyses. Rare allele filtering is a common method for diversity and population structure analysis.<br>For structure analysis, D. longiflora samples were excluded because they did not provide additional information.<br>Three D. longiflora samples that are genetically far from D. exilis samples were excluded in smc++ analyses as well as unusable SNPs were excluded according to msmc-tools advices by excluding SNPs from regions that are not "callable" (e.g. repeated regions). msmc-tools was used to create a mask for excluding these SNPs from all smc++ analyses.

Replication	For DeSh1 phenotyping experiment, shattering was tested based on three panicles from each plant to assure that results are valid and reliable. All attempts at replication were successful.<br>For oligo painting FISH experiment of homoeologous chromosomes, each oligo painting FISH experiment was repeated independently at least two times.<br>For the FISH experiment to identify centromeric repeat of Digitaria exilis, experiment has been repeated independently three times.
Randomization	Panicles for DeSh1 phenotyping were taken from different corresponding accessions that were grown in greenhouse and distributed randomly in the glass room
Blinding	Blinding was not relevant to our study, as the study involves plant genome sequencing and re-sequencing data for population analyses

Reporting for specific materials, systems and methods

We require information from authors about some types of materials, experimental systems and methods used in many studies. Here, indicate whether each material, system or method listed is relevant to your study. If you are not sure if a list item applies to your research, read the appropriate section before selecting a response.

Materials & experimental systems

n/a	Involved in the study
☒	☐ Antibodies
☒	☐ Eukaryotic cell lines
☒	☐ Palaeontology
☒	☐ Animals and other organisms
☒	☐ Human research participants
☒	☐ Clinical data

Methods

n/a	Involved in the study
☒	☐ ChIP-seq
☒	☐ Flow cytometry
☒	☐ MRI-based neuroimaging
