# Supplementary material for: Fonio millet genome unlocks African orphan crop diversity for agriculture in a changing climate
Source: Nat Commun. 2020 Sep 8;11:4488. doi: 10.1038/s41467-020-18329-4 (PMC7479619; doi:10.1038/s41467-020-18329-4)
Supplement: Supplementary file 4 — Description of Additional Supplementary Files [file 41467_2020_18329_MOESM4_ESM.docx]

**Description of Additional Supplementary data files**

File Name: Supplementary Data 1
Description: Synteny analysis between *Digitaria exilis* and *Setaria italica*, *Panicum miliaceum*, *Panicum hallii*, *Sorghum bicolor, Zea mays*, *Oryza sativa*, *Brachypodium distachyon*, *Hordeum vulgare*, *Aegilops tauchii*, *Triticum aestivum*

File Name: Supplementary Data 2

Description: Reconstruction of the hypothetical ancestral genomic state of the Paniceae. The syntenic-block markers are ordered in columns for each chromosome group of each species. *Digitaria exilis* sub-genome B (DexiB) was selected as the 'reference' genome and syntenic-block markers are numbered according the the reference. If the syntenic-block marker is reversed in comparison to the reference it is indicated by a minus. DexiA = *D. exilis* sub-genome A, Pha = *P. hallii*, Pmi1 = *P. miliaceum* sub-genome 1, Pmi2 = *P. miliaceum* sub-genome 2, Set = *S. itali*ca. The *P. miliaceum* sub-genomes are not completely phased and comparisons can only be done within a chromosome group. The other sheets list all the orthologous relationships for each chromosome group. Syntenic blocks and ancestral genes (genes present in each species or sub-genomes) are indicated in the two first columns.

File Name: Supplementary Data 3

Description: Estimation of expression per transcript using the RSEM pipeline

File Name: Supplementary Data 4

Description: Number of raw reads, total base pairs (Gb) and sequence coverage of *Digitaria exilis* and *Digitaria longiflora*. Coverage was calculated based on the CM05836 assembly size. Samples in blue were excluded from analyses because of missing data.

File Name: Supplementary Data 5

Description: Mapping rate of raw reads of each re-sequenced *Digitaria exilis* and *Digitaria longiflora* accession to the CM05836 reference assembly. Samples in blue were excluded from analyses because of missing data.

File Name: Supplementary Data 6

Description: Passport information of *Digitaria exilis* and *Digitaria longiflora* accessions. Included are accession name, country of origin, latitude (Lat.), longitude (Long.), altitude (Alt.), mean temperature of the wettest quarter (Mean temp.), mean precipitation of the wettest quarter (Mean prec.), ethnicity, linguistic groups and genetic cluster (based on *K*=6 and 70% ancestry threshold)

File Name: Supplementary Data 7

Description: Candidate SNPs, candidate loci, and candidate genes for GWAS with bioclimatic and ethnic data

File Name: Supplementary Data 8

Description: Candidate regions and candidate genes under selection
