# Supplementary material for: Fonio millet genome unlocks African orphan crop diversity for agriculture in a changing climate
Source: Nat Commun. 2020 Sep 8;11:4488. doi: 10.1038/s41467-020-18329-4 (PMC7479619; doi:10.1038/s41467-020-18329-4)
Supplement: Supplementary file 2 — Peer Review [file 41467_2020_18329_MOESM2_ESM.pdf]

Reviewer #1 (Remarks to the Author):

Review of NCOMMS-20-14724-T

Here, Abrouk and colleagues develop a short-read based assembly and annotation for the orphan crop fonio, and present a highly detailed set of accompanying population (and to a lesser degree quantitative) genomics resources and analyses. The narrative is very well written, the methods are mostly well laid out, and the scope and scale of result interpretations and discussion are in line with the data and methods used. I have a number of minor and not-so-minor comments and suggestions, which I lay out below.

Genome assembly. As far as short-read based assemblies go, this is a pretty good one. I appreciate the multiple lines of evidence used for contig integration (bionano/Hi-C). The only minor comment I have relates to lines 115-116. Just because there is no diploid ancestor doesn't mean you can't phase the subgenomes (or is this not what you mean?) … either way, for an example of how this can be done, see VanBuren's recent study in Teff (https://www.nature.com/articles/s41467-020-14724-z).

Annotation. I think it needs to be stated up front that you don't have any root-tissue in your rnaseq samples, nor any stress conditions. I don't think this is a huge problem, but might be why you see fewer genes and homeologs than might be expected. Also, it is not clear how you define homeologs / orthologs … is it really just on high blast scores (line 638-639)? I'd expect not, since in grasses, this will turn up a whole ton of paralogs from the Rho WGD.

Resequencing. I didn't see anywhere in the methods a detailed description of DNA sequencing (e.g. depth, technology, lib construction, etc.) or extraction, other than a cursory mention (line 407-408). Am I missing something? Also, I'm a bit surprised at the extremely high level of intergenic SNPs. Were SNPs near repeats / indels masked or treated differently in some way? Please add a note to the methods about this, as it is typical and important to ignore snps near large indels and in repeats. I just worry that maybe the repeat-rich and gene-poor pericentromeres might be driving a lot of this signal. Also, if you sequenced 166 genotypes and 44% of all the sites had MAF < 0.01, then you are saying almost half of all SNPs are due to a private allele in a single library. This is highly suspect, and I'd be concerned that these are not real. Please provide some explanation about why you kept these, and why you think a whopping 44% of all sites have alleles private to a single accession.

Do lines 202-205 state that climate is significant while controlling for geography (e.g. partial mantel test) or that it is significant on its own? If the former, please state the partial mantel r. If the latter, then I'd highly advise doing partial tests instead, as climate and geography are usually correlated.

There are a number of places that demand statistical support. Even places where a P-value is assigned, there are no details about the df, statistical test, etc (e.g. lines 203, 207, 208, …). This is crucial and needs to be resolved. Here is a non-exhaustive list: subgenome expression bias (line 157), comparison of two LD decay rates that look superficially similar (line 182), comparison of genetic distance (line 187) …

I'm having trouble understanding how you genotyped a 60kb deletion with short reads … it is not outlined in the methods. I imagine that low read coverage is the statistic, but this can be highly error prone. For example, just using low unique-mapping coverage over a region wouldn't be appropriate unless you could show that there is no similar sequence in the rest of the genome that caused multimappers.

Line 601 – how was devtools used to get correlation coefs? I think this is miss-referenced.

I'm a bit surprised there wasn't more made out of the phylogenetic position of Digitaria … as a root to the Panicoideae, it could provide a really nice anchor for phylogenomic and ancestral state

reconstruction of the many very high quality Panicoideae genomes.

John T. Lovell
7-May 2020

Reviewer #2 (Remarks to the Author):

This manuscript describes the results obtained during the development of important genomic resources of Fonio, an important African crop that may play an important role in drylands. In particular the authors present the de novo assembly of a reference genome and the resequencing of 183 wild and domesticated cultivated genotypes. While, these results will have an important implication for crop breeding the paper is mostly descriptive and without major outcomes. Moreover, the manuscript it is not well written often with confusion between the different sections (introduction, material and methods, results, and discussion) that makes it hard to follow. For these reasons I do not think that the submitted manuscript is of a sufficient quality to meet the standards for publication in a scientific journal.

Reviewer #3 (Remarks to the Author):

Abrouk et al. provide a new genome assembly resource for fonio millet (Digiteria exilis) along with genomic resources from resequenced accessions. The genome assembly provides a chromosome scale annotated references based upon a host of next generation sequencing libraries, along with integration using Hi-C and bionano optical maps. I am not an expert on genome assemblies, but it seems a solid resource that will be useful and provides another valuable genome for comparative genomics in the grasses.

In addition to the genome assembly, the manuscript provides an analysis of the polyploid nature of fonio millet, a comparison of subgenome dominance, and an analysis of diversity and population structure from a set of resequenced collections. Each of these analyses is relatively straightforward and descriptive, and for the most part provides expected results. Fornia millet is a relatively recent allopolyploid which has only minor subgenome dominance. The population genetic analyses provide insight into the geographical distribution of variation, and allow some statements about the impacts of domestication on genetic diversity. One interesting set of analyses explore relationships between climate of origin, geographical space, and local cultural groups on millet diversity – the idea here is to try and disentangle natural versus human use impacts on the spatial distribution of genetic diversity. The manuscript provides statistical evidence for correlations between climate/ethic groups and genetic variation, but there is little interpretation or insight gained from the analyses. Are specific types of genes driving these associations? Can these analyses provide insight into candidate genes (or biological processes) underlying adaptation? Or that were deferentially selected by ethnic groups?

The final component of the paper focuses on detected footprints of adaptation and domestication by looking at outlier genomic regions for three metrics – a site frequency spectrum test – patterns of nucleotide diversity – and genetic differentiation as measured by Fst. Here, candidate regions were selected as occurring in top 1% tail of the distribution. The authors report on 78 (CLR test), diversity (311), and Fst (208) regions of interest. It's important to note that there will always be outliers, and so there are always candidate genes to talk about from this type of analysis. It might also be worth noting that many non-selective processes and demographic events can generate outlier loci. They note several well-known domestication genes, especially those involved in seed shattering, in their lists. These analyses point to some interesting candidates for subsequent analyses but by no means are proof of their phenotypic effect or role in adaptation/domestication. But, they are valuable hypotheses in a non-model orphan crop.

The text of the manuscript reads well. I think it can be improved by providing a bit more background on fonio millet – background on its origins, mating system, or even more information about its domestication. Not many plant biologists will be familiar with fornio and as such the manuscript may not attract a broad audience.

Point-by-point response to reviewers

Reviewer #1 (Remarks to the Author):

Review of NCOMMS-20-14724-T

Here, Abrouk and colleagues develop a short-read based assembly and annotation for the orphan crop fonio, and present a highly detailed set of accompanying population (and to a lesser degree quantitative) genomics resources and analyses. The narrative is very well written, the methods are mostly well laid out, and the scope and scale of result interpretations and discussion are in line with the data and methods used. I have a number of minor and not-so-minor comments and suggestions, which I lay out below.

Genome assembly. As far as short-read based assemblies go, this is a pretty good one. I appreciate the multiple lines of evidence used for contig integration (bionano/Hi-C). The only minor comment I have relates to lines 115-116. Just because there is no diploid ancestor doesn't mean you can't phase the subgenomes (or is this not what you mean?) … either way, for an example of how this can be done, see VanBuren's recent study in Teff

<u>Our response</u>: Similar to what was done in Teff, we used a bioinformatics approach based on repeats to phase the two sub-genomes (Fig. 2b). This approach, however, is very different from using genomic information of clear diploid ancestors as it was for example done for tetraploid quinoa (Jarvis et al. 2017 Nature 542, 307-312). This is what we want to express here. We have slightly modified the statement as follows: (lines 121-123; line numbers refer to the manuscript version with track changes) 'Without a clear diploid ancestor, we could not directly disentangle the two sub-genomes based on genomic information from diploid ancestors. We thus used a genetic structure approach based on full-length long terminal repeat retrotransposons (fl-LTR-RT) as an alternative strategy'.

Annotation. I think it needs to be stated up front that you don't have any root-tissue in your rnaseq samples, nor any stress conditions. I don't think this is a huge problem, but might be why you see fewer genes and homeologs than might be expected. Also, it is not clear how you define homeologs / orthologs … is it really just on high blast scores (line 638-639)? I'd expect not, since in grasses, this will turn up a whole ton of paralogs from the Rho WGD.

<u>Our response:</u> True, our RNA-Seq samples did not include tissues from roots and stress conditions. It is difficult to judge if this affected the number of predicted gene models or not. In addition to ab initio gene prediction we also included evidence-based approaches to obtain optimal results. The number of gene models annotated in fonio (59,844) is comparable to tetraploid grass genomes of similar size, e.g. broomcorn millet (55,930 gene models) and teff (68,255 gene models). We do not think that we annotated fewer genes than might be expected for a grass genome. The number of homoeologs/orthologs is rather low because we used very stringent criteria to define them, which is important for the Ks analyses. Homoeologs/orthologs were not simply defined based on highest BLAST scores. We used a synteny-based approach in three steps to define homoeologs/orthologs. (1) all-versus-all protein comparisons using BLAST (E-value $10e^{-5}$), followed by (2) pairwise block detection using MCScanX , and (3) postprocessing

of the collinear blocks in order to retrieve only 1:1 homoeologous relationships. Parameter settings for MCScanX have been tested with different setting as describe in Zhao and Schranz (2019 PNAS 116:2165-2174) and we adopted '-m25 -s10' (-s: number of minimum matched syntenic anchors, -m: number of max gene gaps). For the postprocessing, we decided to only retain 1:1 relationships (1:2 for maize and broomcorn millet because their individual sub-genomes are not clearly defined). To make this clearer, we have modified the methods section as follows (lines 564-575): 'To identify syntenic relationships, BLASTP (E-value 1x10^{-5}) was first used to perform all-versus-all protein comparisons between CM05836 and Setaria italica, Panicum miliaceum, Panicum hallii, Sorghum bicolor, Zea mays, Oryza sativa, Brachypodium distachyon, Hordeum vulgare, Triticum aestivum and Aegilops tauschii. Then, MCScanX (-m 25; -s 10) was used for pairwise synteny block detection. For all the comparisons, fonio sub-genomes A and B were analysed independently and only 1:1 relationships were retained (1:2 relationships for tetraploid broomcorn millet and the ancient tetraploid maize, where individual sub-genomes are not clearly phased).'

Resequencing. I didn't see anywhere in the methods a detailed description of DNA sequencing (e.g. depth, technology, lib construction, etc.) or extraction, other than a cursory mention (line 407-408). Am I missing something? Also, I'm a bit surprised at the extremely high level of intergenic SNPs. Were SNPs near repeats / indels masked or treated differently in some way? Please add a note to the methods about this, as it is typical and important to ignore snps near large indels and in repeats. I just worry that maybe the repeat-rich and gene-poor pericentromeres might be driving a lot of this signal. Also, if you sequenced 166 genotypes and 44% of all the sites had MAF < 0.01, then you are saying almost half of all SNPs are due to a private allele in a single library. This is highly suspect, and I'd be concerned that these are not real. Please provide some explanation about why you kept these, and why you think a whopping 44% of all sites have alleles private to a single accession.

<u>Our response:</u> *A paragraph describing the DNA sequencing has been added to the methods section as follows (lines 592-615): 'One or two young leaves from D. exilis or D. longiflora seedlings were collected in 2 ml Eppendorf tubes, flash-frozen in liquid nitrogen, and ground with glass beads in a SPEX SamplePrep Geno/Grinder 2010. Samples were mixed with 1.2 ml 2x CTAB buffer (2% CTAB, 200 mM Tris/HCl (pH 8), 20 mM EDTA, 1.4 M NaCl, 1.0% PVP, 0.28 M β-mercaptoethanol) and incubated at 65°C with periodic mixing for 60 minutes. Samples were cooled to room temperature and centrifuged at 2,500 rpm for 10 minutes. Subsequently, 900 μl of the supernatant was transferred to a fresh 2 ml Eppendorf tube and incubated with 800 μl dichloromethane:isoamyl alcohol (24:1) in an overhead-shaker at half-speed at 4°C for 15 minutes. After centrifugation at 13,000 rpm for 15 minutes, 800 μl of the supernatant was transferred to a fresh 2 ml Eppendorf tube and incubated with 5 μl RNase A (10 mg/ml, EN0531, ThermoFisher Scientific) at 37°C for 15 minutes. DNA was precipitated by adding 560 μl isopropanol and mixing the tubes by inversion. Precipitated DNA was pelleted by centrifugation at 4°C and 13,000 rpm for 10 minutes and the supernatant discarded. The DNA pellet was washed in a first ethanol wash (76% ethanol, 200 mM sodium acetate) for 15 minutes and in a second ethanol wash (76% ethanol, 10 mM ammonium acetate) for five minutes. Subsequently, DNA pellets were air-dried to remove residual ethanol and eluted in 50 μl TE buffer (10 mM Tris/HCl (pH 8), 1 mM EDTA). Extracted DNA was quantified with the Qubit dsDNA HS Assay (Q32851, ThermoFisher Scientific), the purity was confirmed by checking 260/280 and 260/230 ratios on a Nanodrop spectrophotometer, and the integrity was confirmed by analyzing 1 μl per sample on a 1% TAE agarose gel. Library preparation and*

sequencing were performed by Novogene. Briefly, 1.0 µg DNA per sample was used as input material for the DNA sample preparations. Sequencing libraries were generated using the NEBNext® Ultra II DNA Library Prep Kit following manufacturer's instructions. Libraries were sequenced using an Illumina NovaSeq 6000 system.'

Thirty-five percent of the intergenic SNPs are gene-proximal (2 kb up- or downstream of coding sequence). Our fraction of 'true' intergenic SNPs is thus comparable to many other studies (see for example Wang et al 2019 Genome Biology 20:74 and Liang et al 2019 Nature Communications 10:1190). We have updated the corresponding statement as follows (lines 186-188): Approximately 30.2% of the SNPs were gene-proximal (2 kb up- or downstream of a coding sequence), 9.5% in introns and 6.2% in exons.

The relatively high number of rare variants is caused by a few genetically distant Digitaia longiflora accessions. For example, 84% of all private SNPs were present in the 14 D. longiflora accessions. We have indicated this in the main text as follows (lines 191-193): 'Forty-four percent of the total SNPs (4,901,160 SNPs) were rare variants with a minor allele frequency of less than 0.01 (Supplementary Fig. 12). The vast majority of the rare variants was found in a few very diverse D. longiflora accessions.' Private SNPs were excluded from the PCA and Structure analyses.

We have chosen a robust SNP filtering approach that was used in previous studies (Cubry et al. 2018 Current Biology 28:2274-2282 and Burgarella et al. 2019 Nature Ecology & Evolution 2: 1377-1380). In particular, filtering for SNP clusters (Supplementary Table 12) removes most SNPs that are possibly caused by wrongly mapped reads to repeats. We added the new supplementary figure 11a that shows the genetic divergence of the re-sequenced D. exilis accessions from the CM05836 reference. The figure clearly shows that the re-sequenced CM05836 sample had by far the lowest genetic divergence. In the main text, we refer to this supplementary figure as follows (lines 181-184): 'The error rate of variant calling (proportion of segregating sites in a re-sequenced CM05836 sample compared to the reference assembly) was 0.04%, which is comparable to other studies. The re-sequenced CM05836 sample showed the lowest genetic divergence from the reference assembly of all re-sequenced accessions (Supplementary Fig. 11a).'

Do lines 202-205 state that climate is significant while controlling for geography (e.g. partial mantel test) or that it is significant on its own? If the former, please state the partial mantel r. If the latter, then I'd highly advise doing partial tests instead, as climate and geography are usually correlated.

<u>Our response</u>: *The initial tests were done for climate and geography independently. As supplementary figure 15 shows, however, climate and geography are correlated. We thus included a partial Mantel test to test for a correlation between the genetic distance matrix and climate while controlling for geography. The statement in the main text has been modified as follows (lines 215-225): 'We observed a significant correlation (Pearson's correlation; $p < 0.05$, $df = 155$) between the genetic population structure (first principal component of PCA) and climate (i.e. mean temperature and precipitation of the wettest quarters) as well as geography (i.e. latitude, longitude and altitude) (Fig. 3b, Supplementary Fig. 15, Supplementary Table 8). The relationship between genetic differentiation (genetic distance matrix) and climate was still significant when accounting for geographic distance (partial Mantel test; Mantel r = 0.30; p = 0.001). A significant correlation was also observed between the genetic distance matrix and the dissimilarity matrices of ethnic and linguistic groups (fisher test; p = 0.0005, Mantel test; Mantel r ethnic*

= -0.19; Mantel r linguistic = -0.13; p = 0.001). The effect of ethnic groups remained significant even when we controlled for geographic and climatic factors (ANCOVA; p = 0.045; df = 31) (Supplementary Table 9).'

There are a number of places that demand statistical support. Even places where a P-value is assigned, there are no details about the df, statistical test, etc (e.g. lines 203, 207, 208, …). This is crucial and needs to be resolved. Here is a non-exhaustive list: subgenome expression bias (line 157), comparison of two LD decay rates that look superficially similar (line 182), comparison of genetic distance (line 187)

Our response: *Statistical tests have been added and details about df and statistical tests used have been included. In particular, we have added the new supplementary figure 14b that illustrates the genetic differences between D. exilis and D. longiflora.*

I'm having trouble understanding how you genotyped a 60kb deletion with short reads … it is not outlined in the methods. I imagine that low read coverage is the statistic, but this can be highly error prone. For example, just using low unique-mapping coverage over a region wouldn't be appropriate unless you could show that there is no similar sequence in the rest of the genome that caused multimappers.

Our response: *The 60 kb deletion was initially identified as absence of read mapping. We fully agree with the reviewer that this approach is error prone. We have thus developed a robust PCR-based haplotype marker. The haplotype marker experimentally confirmed the 60 kb deletion. We have added the following sentence in the main text (line 276-278): 'This deletion was identified by the lack of mapped reads and was confirmed by PCR amplification and Sanger sequencing.'. The primer sequences are given in the methods section (lines 735-741): 'Two specific primer pairs were designed to confirm the 60 kb deletion. The first primer pair amplifies a ̴1 kb fragment within the DeSh1-9A gene (forward primer 5'-GTGACCATGCCAAGCAGGCG-3'; reverse primer 5'-GCTAGCTTGAGGTATTTACGG-3'). The second primer pair was designed in the flanking region of the deletion and amplifies a ̴750 bp fragment in accessions that carry the deletion (forward primer 5'-GCCGTATATAGTTGCCGCATCA-3'; reverse primer 5'-CCTACCGTTAGATCCGTGCGGA-3').'*

Line 601 – how was devtools used to get correlation coefs? I think this is miss-referenced.

Our response: *This was indeed a mistake and the stats package was used. Thanks a lot for pointing this out.*

I'm a bit surprised there wasn't more made out of the phylogenetic position of Digitaria … as a root to the Panicoideae, it could provide a really nice anchor for phylogenomic and ancestral state reconstruction of the many very high quality Panicoideae genomes.

Our response: *We used an approach based on synteny blocks to infer the ancestral state of the Paniceae. This approach also revealed lineage specific structural rearrangements, deletions, and translocations. The results are summarized in the new Supplementary data 2. In the main text, we refer to this data set as follows (lines 156-158): 'The phylogenetic position of D. exilis as a basal taxon of the Paniceae allowed us to reconstruct the hypothetical ancestral genomic state of this tribe (Supplementary data 2).' In the*

methods section, we describe our approach as follows (lines 581-586): 'We used the Maximum Likelihood for Gene-Order Analysis (MLGO) tool to reconstruct the hypothetical ancestral genome of the Paniceae tribe from syntenic-block data of D. exilis, S. italica, P. miliaceum, and P. hallii. Syntenic blocks were identified using MCScanX using the B sub-genome of D. exilis as 'reference'. Then, syntenic blocks were combined into 'syntenic-block markers' as described previously. Syntenic-block markers along with the known species tree were provided as inputs to the MLGO server to infer the ancestral state of the Paniceae.'

Reviewer #2 (Remarks to the Author):

This manuscript describes the results obtained during the development of important genomic resources of Fonio, an important African crop that may play an important role in drylands. In particular the authors present the de novo assembly of a reference genome and the resequencing of 183 wild and domesticated cultivated genotypes. While, these results will have an important implication for crop breeding the paper is mostly descriptive and without major outcomes. Moreover, the manuscript it is not well written often with confusion between the different sections (introduction, material and methods, results, and discussion) that makes it hard to follow. For these reasons I do not think that the submitted manuscript is of a sufficient quality to meet the standards for publication in a scientific journal.

Reviewer #3 (Remarks to the Author):

Abrouk et al. provide a new genome assembly resource for fonio millet (Digiteria exilis) along with genomic resources from resequenced accessions. The genome assembly provides a chromosome scale annotated references based upon a host of next generation sequencing libraries, along with integration using Hi-C and bionano optical maps. I am not an expert on genome assemblies, but it seems a solid resource that will be useful and provides another valuable genome for comparative genomics in the grasses.

In addition to the genome assembly, the manuscript provides an analysis of the polyploid nature of fonio millet, a comparison of subgenome dominance, and an analysis of diversity and population structure from a set of resequenced collections. Each of these analyses is relatively straightforward and descriptive, and for the most part provides expected results. Fornia millet is a relatively recent allopolyploid which has only minor subgenome dominance. The population genetic analyses provide insight into the geographical distribution of variation, and allow some statements about the impacts of domestication on genetic diversity. One interesting set of analyses explore relationships between climate of origin, geographical space, and local cultural groups on millet diversity – the idea here is to try and disentangle natural versus human use impacts on the spatial distribution of genetic diversity. The manuscript provides statistical evidence for correlations between climate/ethic groups and genetic variation, but there is little interpretation or insight gained from the analyses. Are specific types of genes driving these associations? Can these analyses provide insight into candidate

genes (or biological processes) underlying adaptation? Or that were deferentially selected by ethnic groups?

<u>Our response</u>: *We addressed this point by performing genome-wide association studies (GWAS) with bioclimate and ethnic data as inputs followed by GO term enrichment analyses. The results are summarized in the new supplementary figures 16 and 17 as well as in the new supplementary data 4. While these analyses provide some insight into possible biological processes and candidate genes we need to cautious with their interpretation. We have summarized the results in the main text as follows (lines 225-239; line numbers refer to the manuscript version with track changes): 'Association analyses based on climate variables revealed 38 and 179 loci that might be involved in adaptation to mean temperature and mean precipitation of the wettest quarters, respectively (Supplementary Fig. 16, Supplementary data 4). Loci associated with temperature contained genes enriched for functions related to hormone metabolic processes, hormone biosynthesis, carbohydrate metabolic processes, homeostatic process, plant development processes, and plant growth (shoot apical meristem maintenance, root growth and development) (Supplementary data 4). Similarly, genes associated with precipitation were enriched in functions related to plant development and growth (Supplementary data 4). A genome-wide association study with ethnic groups revealed significant associations for 55 and 227 SNPs with Bambara and Fula ethnic groups, respectively (Supplementary Fig. 17, Supplementary data 4). In particular, there were three prominent peaks on chromosomes 2A, 2B, and 6A. The peaks on chromosomes 2A and 2B fell into a region that contained a homolog of the Arabidopsis WSD1 wax ester synthase/diacylglycerol acyltransferase gene. WSD1 is involved in accumulation of waxes under drought stress, possibly indicating selection for adaptation to drought by certain ethnic groups.' The methods section has been updated as follows (lines 669-699): Synthetic climate variables were retrieved from the WorldClim v1.4 database. We focused our analysis on two climate variables that were the most pertinent to an annual species, i.e. mean temperature of the wettest quarter (bio8) and mean precipitation of the wettest quarter (bio 16), respectively. A GWAS with these climate variables was then performed following the pipeline and steps described in Cubry et al. (2020). Briefly, we first use the impute method from the package LEA to fill the gaps in the genotypic matrix in order to achieve greater power. We then filtered out SNPs with a MAF below 5 %. We used three different models of association to perform the GWAS, the EMMA, Gapit and LFMM models. For Gapit, we considered 6 principal components, which we chose from the previously described structure analysis. The LFMM v2 method implements a Latent Factor Mixed Model that estimates unknown confounding factors (the latent factors) jointly using the genotypic and phenotypic matrices. For this specific model, the estimation of latent factors was performed using both the phenotypic data (i.e. the climate variable in consideration) and a genotypic matrix of SNPs with a MAF higher than 20 %. Once the GWAS step was performed, we used a FDR approach to estimate the significance of the associations for each analysis, retaining a FDR threshold of 5 %, resulting in a list of candidate positions for each variable and each GWAS method. We then considered a genomic window of 50 kb upstream and 50 kb downstream for each position as a candidate region, which we denominated Quantitative Trait Locus (QTL). When two QTLs overlapped, they were combined.*

To test SNP associations to ethnolinguistic groups, we used the PLINK software (v1.90). We filtered out the SNPs with MAF below 5%, more than 10% missing data, and in linkage disequilibrium, resulting in 897,796 SNPs. The association of SNPs with each ethnic and linguistic group was tested by a logistic regression model assuming additive genetic effects, using three principal components as covariates to control for population stratification. Significant associations were identified above the threshold of P value = $1e^{-05}$*. Manhattan plots were plotted to display the associations using the 'qqman' R package.*

GO terms for all fonio protein sequences were assigned using the DeepGOPlus model. We only considered GO labels that have a confident score > 0.3. Enrichment analysis for GO biological processes, cellular components, and molecular functions of the genes of interests from climate association analyses was performed using both classic and weight01 methods implemented in the R package TopGO.

The final component of the paper focuses on detected footprints of adaptation and domestication by looking at outlier genomic regions for three metrics – a site frequency spectrum test – patterns of nucleotide diversity – and genetic differentiation as measured by Fst. Here, candidate regions were selected as occurring in top 1% tail of the distribution. The authors report on 78 (CLR test), diversity (311), and Fst (208) regions of interest. It's important to note that there will always be outliers, and so there are always candidate genes to talk about from this type of analysis. It might also be worth noting that many non-selective processes and demographic events can generate outlier loci. They note several well-known domestication genes, especially those involved in seed shattering, in their lists. These analyses point to some interesting candidates for subsequent analyses but by no means are proof of their phenotypic effect or role in adaptation/domestication. But, they are valuable hypotheses in a non-model orphan crop.

The text of the manuscript reads well. I think it can be improved by providing a bit more background on fonio millet – background on its origins, mating system, or even more information about its domestication. Not many plant biologists will be familiar with fornio and as such the manuscript may not attract a broad audience.

<u>Our response:</u> *We have provided additional background information on fonio millet in the introduction as follows: (lines 68-70) 'Fonio is a small annual herbaceous C4 plant, which produces very small (~1 mm) grains that are tightly surrounded by a husk (Fig. 1).' and lines 78-83 'The most likely wild progenitor of cultivated fonio is the tetraploid annual weed D. longiflora that is widely distributed in tropical Africa. It has been suggested that fonio was domesticated more than 5,000 years ago in the Inner Niger Delta of central Mali. Compared to D. exilis, the wild D. longiflora shows even heavier seed shattering and hairy spikelets, which suggests that some selection for reduced seed shattering and spikelet hairiness occurred in fonio.' The reproductive system of fonio is mentioned in the first sentence of the result section (line 93): 'Fonio is a tetraploid species (2n = 4x = 36) with a highly inbreeding reproductive system.'*

REVIEWERS' COMMENTS:

Reviewer #1 (Remarks to the Author):

The authors have satisfactorily addressed my concerns in their response to reviews. I only have one gripe: I don't believe there is a statement in the actual text about which loci where used in the popgen analyses. Something to the effect of what was stated in the r2r would suffice: 'Private SNPs were excluded from the PCA and Structure analyses'.

Cheers,
John Lovell
9-July 2020

Reviewer #3 (Remarks to the Author):

Abrouk et al. provide a genome assembly and associated genomic analyses of Fonio millet, including analyses of genetic diversity and the identification of a number of candidate genes. The manuscript is an improved revision that clarified a number of points raised by reviewers, expands several analyses, and provides an important resource for comparative biology. The authors have responded to all of the questions from the earlier review, and I have no new questions.

REVIEWERS' COMMENTS:

Reviewer #1 (Remarks to the Author):

The authors have satisfactorily addressed my concerns in their response to reviews. I only have one gripe: I don't believe there is a statement in the actual text about which loci where used in the popgen analyses. Something to the effect of what was stated in the r2r would suffice: 'Private SNPs were excluded from the PCA and Structure analyses'.

Our response: *We have added the following sentence in the main text (line 675): 'i.e., private SNPs were excluded'*

Reviewer #3 (Remarks to the Author):

Abrouk et al. provide a genome assembly and associated genomic analyses of Fonio millet, including analyses of genetic diversity and the identification of a number of candidate genes. The manuscript is an improved revision that clarified a number of points raised by reviewers, expands several analyses, and provides an important resource for comparative biology. The authors have responded to all of the questions from the earlier review, and I have no new questions.
